# Use of homologous and heterologous gene expression profiling tools to characterize transcription dynamics during apple fruit maturation and ripening

**DOI:** 10.1186/1471-2229-10-229

**Published:** 2010-10-25

**Authors:** Fabrizio Costa, Rob Alba, Henk Schouten, Valeria Soglio, Luca Gianfranceschi, Sara Serra, Stefano Musacchi, Silviero Sansavini, Guglielmo Costa, Zhangjun Fei, James Giovannoni

**Affiliations:** 1 Department of Fruit Tree and Woody Plant Science, University of Bologna, Viale Fanin 46, 40121 Bologna, Italy; 2 Boyce Thompson Institute for Plant Research, Cornell University Campus, Ithaca, New York, 14853, USA; 3 U.S. Department of Agriculture, Agricultural Research Service, Robert W. Holley Center, Ithaca, New York, 14853, USA; 4 Plant Breeding, Wageningen-UR, Droevendaalsesteeg 1,6700 AA Wageningen, The Netherlands; 5 Dept. of Biomolecular Sciences and Biotechnology, University of Milano, via Celoria 26, 20133 Milano, Italy; 6 IASMA Research and Innovation Centre, Foundation Edmund Mach, Via E. Mach 1, 38010 San Michele all'Adige, Trento, Italy

## Abstract

**Background:**

Fruit development, maturation and ripening consists of a complex series of biochemical and physiological changes that in climacteric fruits, including apple and tomato, are coordinated by the gaseous hormone ethylene. These changes lead to final fruit quality and understanding of the functional machinery underlying these processes is of both biological and practical importance. To date many reports have been made on the analysis of gene expression in apple. In this study we focused our investigation on the role of ethylene during apple maturation, specifically comparing transcriptomics of normal ripening with changes resulting from application of the hormone receptor competitor 1-Methylcyclopropene.

**Results:**

To gain insight into the molecular process regulating ripening in apple, and to compare to tomato (model species for ripening studies), we utilized both homologous and heterologous (tomato) microarray to profile transcriptome dynamics of genes involved in fruit development and ripening, emphasizing those which are ethylene regulated.

The use of both types of microarrays facilitated transcriptome comparison between apple and tomato (for the later using data previously published and available at the TED: tomato expression database) and highlighted genes conserved during ripening of both species, which in turn represent a foundation for further comparative genomic studies.

The cross-species analysis had the secondary aim of examining the efficiency of heterologous (specifically tomato) microarray hybridization for candidate gene identification as related to the ripening process. The resulting transcriptomics data revealed coordinated gene expression during fruit ripening of a subset of ripening-related and ethylene responsive genes, further facilitating the analysis of ethylene response during fruit maturation and ripening.

**Conclusion:**

Our combined strategy based on microarray hybridization enabled transcriptome characterization during normal climacteric apple ripening, as well as definition of ethylene-dependent transcriptome changes. Comparison with tomato fruit maturation and ethylene responsive transcriptome activity facilitated identification of putative conserved orthologous ripening-related genes, which serve as an initial set of candidates for assessing conservation of gene activity across genomes of fruit bearing plant species.

## Background

Fruit are important components in the human diet, serving as sources of vitamins, minerals, fiber and antioxidants [1,2]. In some parts of the world including equatorial regions of Asia, Africa and Americas, fruits can be among the most significant source of limiting nutrients and food security.

During the final stages of development, fruit undergo the unique process of ripening which is often characterized by dramatic changes in color, texture, flavor and aroma, that are controlled by both external signals (light, temperature, hydration) and endogenous hormonal and genetic regulators that render the fruit organ attractive and palatable for seed-dispersing organisms [3].

Based on ripening physiology, apple and tomato are classified as climacteric fruit, in which the onset of ripening is accompanied by a rapid increase in respiration rate, normally coincident with elevated ethylene biosynthesis [4,5]. Ethylene is a gaseous hormone able to trigger and coordinate many physiological and response processes in higher plants, including ripening. In climacteric fruits where the hormone typically plays major regulatory roles [6,7], it has been shown that ethylene dependent and independent events operate in tandem to regulate overall ripening [8,9].

The fact that apple and tomato are climacteric suggests that at least some of the regulatory aspects of ripening in both species may be conserved. Tomato has served as a primary model of ripening research due to its short life cycle, ease of transformation, well characterized germplasm http://tgrc.ucdavis.edu/ and availability of extensive molecular resources http://solgenomics.net; http://ted.bti.cornell.edu/;  http://www.pgb.kazusa.or.jp/kaftom/. In this regard it is an optimal reference system for comparative genomics of climacteric ripening with apple.

In an effort to define comprehensive transcriptome variation with the final aim of identifying candidate ripening genes important for apple and conserved among climacteric species, we employed an expression profiling strategies using both heterologous (HET: tomato) and homologous (HOM: apple) expression platforms. The HET array TOM1 was specifically constructed for functional study in tomato http://ted.bti.cornell.edu/cgi-bin/TFGD/array/home.cgi, while the HOM array was dedicated for apple analysis as a component of the HiDRAS EU-project http://www.hidras.unimi.it. Microarray technology has received enormous emphasis in recent years by the scientific community, due to its capabilities of analyzing transcription activity in a high throughput fashion [10], especially in those species where large amounts of gene sequence are available [11]. We chose to study the ripening process of apple performing a biological assay with an heterologous tomato array because of its large and well detailed collection of genomic information, and because of the success of a similar approach described in both vertebrate species [12] and plants [13,14].

This study aimed to improve knowledge about ripening control in apple by identifying new elements involved in this process, keeping in mind that the use of a heterologous cDNA array is limited to those genes that retain a minimal degree of sequence homology. However, a similar phenomena occurs in cDNA homologous array hybridization, due to the cross hybridization of members belonging to the same gene family characterized by high sequence similarity [15]. The use of TOM1 was justified by its greater coverage, while the value of the HOM apple array was grounded in the fact that this is a fruit dedicated array, already used to identify genes differentially expressed during fruit development and maturation in apple [16], and in this context represented a valuable tool to confirm the heterologous data. The use of a common reference genomic tool is an attractive prospect for analysis of non-model plants. The two platforms were simultaneously assessed to characterize fruit ripening transcription dynamics with an emphasis on ethylene-regulated genes.

Earlier high-throughput genomic efforts on fruit ripening and quality have been reported by several groups, with the first being Aharoni et al. who identified genes related to strawberry fruit quality [17]. With regards to prior transcriptomics studies on tree fruit species, the peach microarray μPEACH1.0 was used to study gene expression changes associated with the transition from pre-climacteric to climacteric fruit development [18], while in nectarine it was used to elucidate transcriptome variation in response to the ethylene perception inhibitor 1-MCP [19].

Apple has been investigated with a small number of microarray platforms, though most are of limited size, or more focused on fruit development and pre-harvest ripening [20-23].

Large scale statistical analysis of ESTs in apple have been reported, including an *in silico *comparison with tomato [24,25]. Gene expression comparisons between apple and tomato, two fleshy-fruited species belonging respectively to the Rosaceae and Solanaceae, could be very informative in unraveling the unique and common determinants of ripening control. Tomato has been widely used as the primary model species for climacteric fruit ripening [1,8,26], and a comprehensive transcriptomic tool kit has been developed to analyze the underlying genetic ripening network. Alba et al. [27,28] described extensive time-series expression profiling of wild-type tomato fruit using the TOM1 array (also used here with apple). In this work [28], 869 of approximately 9,000 genes assayed were differentially expressed during the fruit maturation process, 37% of which were altered in comparison with the *Never ripe *ethylene receptor mutant [29].

Here we report our exploration of ethylene dependent and independent trascriptomics of apple fruit maturation and ripening as compared to tomato. The apple ethylene transcriptome was further characterized in the context of the response caused by the ethylene perception competitor 1-Methylcyclopropene (1-MCP) [30]. We present a comprehensive biological cross-species genomic comparison between apple and tomato, using and comparing homologous and heterologous cDNA microarrays in addition to 2-dimensional protein separation, to highlight conserved and unique gene activities contributing to the complex and important mechanism of climacteric ripening control.

## Methods

### Plant material and characterization of fruit ripening physiology

Mondial Gala fruit were harvested at commercial ripening, and a subset of 40 fruits were treated overnight with 1ppm of 1-MCP at 24°C in sealed containers. Ethylene production was monitored by gas-chromatography (DANI, Monza, Italy), on five fruits, three times/week per two fruit batches, analyzed for 10 and 60 days, respectively, after harvest. In these two time-course experiments, ethylene was measured for both control and treated sample in order to profile a normal evolution compared to the kinetics affected by the ethylene competitor (1-MCP).

Samples were also assessed for fruit firmness with a digital firmness tester (equipped with a 11 mm probe) on the two peeled and opposite fruit surfaces of each fruit tested. Transcriptome profiling was carried out with RNA isolated from seven tissues (collected from the first batch) with three biological replicas for each (total 21 samples). Three time-points spanned early fruit maturation: green (66 DAFB - days after full blossom), breaker (90 DAFB), and red ripe (114 DAFB, also the time of the typical commercial harvest), and four time-points spanned late ripening stages: T1_Ctrl _(120 DAFB) T2_Ctrl _(123 DAFB) for the control, and the two corresponding 1-MCP treated samples: T1_1-MCP _and T2_1-MCP. _The experimental design is thus characterized by two time courses, with the first spanning climacteric ripening (green-breaker-red ripe-T1_Ctrl_-T2_Ctrl_) and the second the same but as influenced by 1-MCP treatment (green-breaker-red ripe-T1_1-MCP_-T2_1-MCP_).

### RNA isolation and HET (heterologous) - HOM (homologous) expression profiling in apple fruit

Total RNA was isolated from liquid nitrogen frozen flesh (stored at -80°C) collected from all stages, using a CTAB-based extraction buffer [31]. After the first precipitation the procedure followed the protocol reported in the TED database http://ted.bti.cornell.edu/cgi-bin/TFGD/array/total_RNA_extraction.cgi and modified [32].

Synthesis and cDNA labelling were performed according to [27]. First-strand synthesis and purification was obtained with the Super-Script™ Indirect cDNA Labelling System Kit (Invitrogen Corp), and microarray hybridization was performed labelling the cDNA with Cy3 for the reference and Cy5 for the experimental test samples. Labelling and hybridization protocols are detailed in the TED database http://ted.bti.cornell.edu/cgi-bin/TFGD/array/total_RNA_label.cgi; http://ted.bti.cornell.edu/cgi-bin/TFGD/array/TOM1_hybridization.cgi [33,34]. Our experimental design employed a common reference design, hybridizing three biological replicates for each time point [35].

### Data Processing

Microarray slides were processed using a two-channel confocal scanner (ScanArray 5000) and the images were acquired and analyzed with ScanArray v3.1 software (Packard Biochip Technologies), setting the PMT at 65-75%, with a scanning resolution of 10 μm. Raw images files were captured and converted to intensity values using Imagene software (v5.6. Bio-Discovery Inc., El Segundo, CA, USA). Data analysis was performed using BRB-Array Tools 3.4 http://linus.nci.nih.gov/BRB-ArrayTools.html, an integrated software package based on R statistic developed by Dr. Richard Simon and Amy Peng Lam [36]. Data were transformed to the log_2 _scale and normalized with the lowess methods to minimize systematic variance. Differentially expressed genes (DEGs) over the time course were identified using the class comparison tool, performing a paired sample t-test (P value < 0.01). The multiple variation tests were used with the maximum false discovery rate set at 0.1 and 90% of confidence.

Expression profile clustering was conduced with the GEPAS web-based resource for microarray gene expression analysis (Gene Expression Profile Analysis Suite, http://gepas.bioinfo.cipf.es [37]). Comprehensive heterologous and homologous data were clustered using the SOTA algorithm (Self Organising Tree Algorithm), an unsupervised neural network with a binary tree topology [38]. Overall graphical representation of the total heterologous transcriptome data was visualized using MATLAB 6.0 (The MathWorks). Microarray data are available in the Array Expression database http://www.ebi.ac.uk/microarray-as/ae/ with the number A-MEXP-1867.

### Quantitative Real Time PCR for gene specific expression profiling

From the seven samples, five μg of total RNA/sample was used to reverse transcribe cDNA using Superscript II Reverse Transcriptase (Invitrogen Technology) with Oligo dT_25_. Real Time PCR was carried out with SYBR^® ^Green PCR Master Mix (Applied Biosystem) using the following primers related to ACO and PG genes: RT-Md-ACO1_for: CAGGCAACGACGCATTCAT, RT-Md-ACO1_rev: GGCGTCCCCAGTTTTCTTCT and RT-Md-PG1_for: ACCGGTGGGATAGCAACATC, RT-Md-PG1_rev: ATTCCCTTTAGCTCCAAAATCGT. Amplicon detection was performed using an ABI Prism 7700 Sequencing Detection System with the following thermal profile: 95°C for 10' and subsequent 40 cycles of 95°C for 15 sec, 60°C for 1 min and 48°C for 30 sec. Amount of target was normalized to an endogenous reference (18S) and expressed as 2^-ΔΔCt ^(Applied Biosystem, User Bulletin #2).

### 2D Proteomic analysis

Total protein extraction used two grams of frozen cortex collected from two samples of Mondial Gala apple: T1_Ctrl _and T1_1-MCP_. The extraction buffer contained 500 mM Tris-HCl (pH 8), 700 mM sucrose, 10 mM EDTA, 4 mM ascorbic acid, 1 mM PMSF, 0.2% Triton X-100, 1 μM leupeptin and 100 mM Pefabloc. Sample were solubilized in 7 M urea, 2 M tiourea, 2% w/v CHAPS, 2% w/v Triton X-100, 2% w/v ampholytes IPG buffer (3-10 pH range), 5% w/v DTT and a trace of bromophenol blue. The first dimension was carried out on an Ettan IPGphor I (Amersham Bioscience) at 70 KVs. The strips were subsequently equilibrated for 12 min in 50 mM Tris-HCl (pH 6,8), 6 M urea, 30% v/v glycerol, 2% w/v SDS and 2% w/v DTT; and for an additional 5 min in 50 mM Tris-HCl (pH 6,8), 6 M urea, 30% v/v glycerol, 2% w/v SDS and 2,5% w/v iodacetamide and a trace of bromophenol blue. After equilibration, the strips were placed on 12.5% polyacrylamide gels (26 cm × 20 cm × 1 mm) in the Ettan Dalt Six Electrophoresis system (Amersham Bioscience), and the second dimension was carried out at 12 mA/gel over-night at 10°C. Gel images were analyzed using the Image Master Platinum v.5.0 Software (Amersham Bioscience). Spot matching was performed using two synthetic gels by overlapping three gels per sample and placing 23 anchors in each gel.

## Results and Discussion

### Transcriptome dynamics in apple fruit

**Figure 1:**
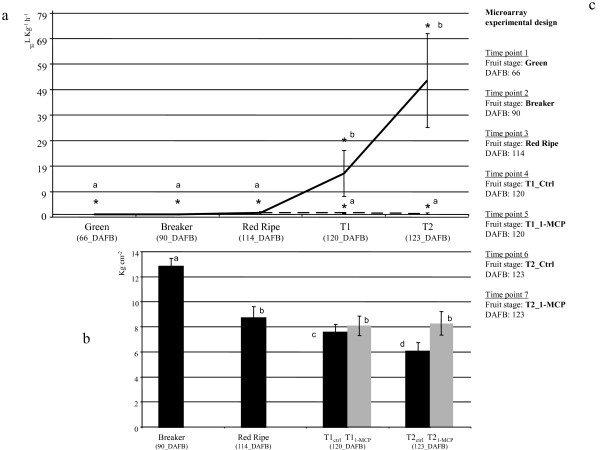
**Ethylene biosynthesis and softening of Mondial Gala apple**. Panel (a) shows ethylene evolution during fruit maturation and ripening. The solid and dashed lines indicate ethylene released by the control samples and the samples treated with 1-MCP, respectively. Asterisks denote samples used in the microarray experiment. Standard error bars are shown; letters denote statistical differences based on ANOVA LSD test (P = 0.05). Panel (b) shows fruit softening during maturation and ripening. Black bars indicate firmness for control samples; grey bars indicate samples treated with 1-MCP. Standard error bars are shown; letters denote samples that are statistically different based on ANOVA LSD test (P = 0.05). Panel (c) lists the samples used in the HET and HOM microarray hybridizations. Abbreviations: Ctrl, control; 1-MCP, 1-Methylcyclopropene.

**Figure 2:**
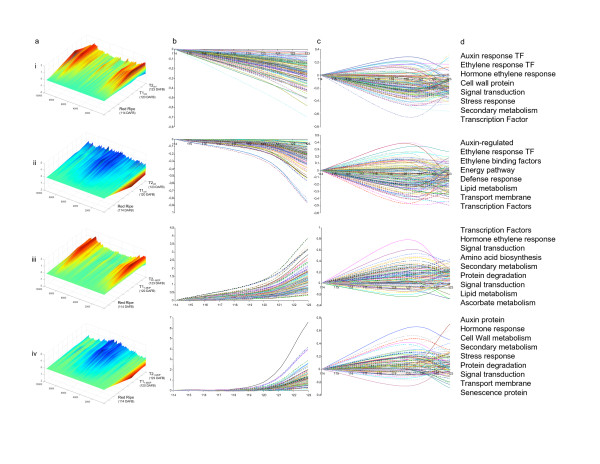
**The effect of 1-MCP on heterologous (HET) transcriptome dynamics during the final stages of apple ripening**. Panel (a) 3D rendering of gene expression between 114 DAFB (red ripe) and 123 DAFB (T2 stage). Red indicates gene expression that is up-regulated; blue indicates gene expression that is down-regulated. Control samples (i and ii) and samples treated with 1-MCP (iii and iv) are shown. The X axis represents unique TOM1 features, the Y axis represents relative expression level after log_2 _transformation, and the Z axis represents time in DAFB. Panel (b) shows four distinct expression profiles identified for the final stages of apple ripening (114 DAFB to 123 DAFB). The X axis represents time (114 DAFB to 123 DAFB) and the Y axis represents relative expression level after log_2 _transformation. Panel (c) shows the effect of 1-MCP on the expression of genes shown in panel (b). Panel (d) lists the annotation categories for genes shown in (b) and (c). Abbreviations: DAFB, days after full bloom; TF, transcription factor.

**Figure 3:**
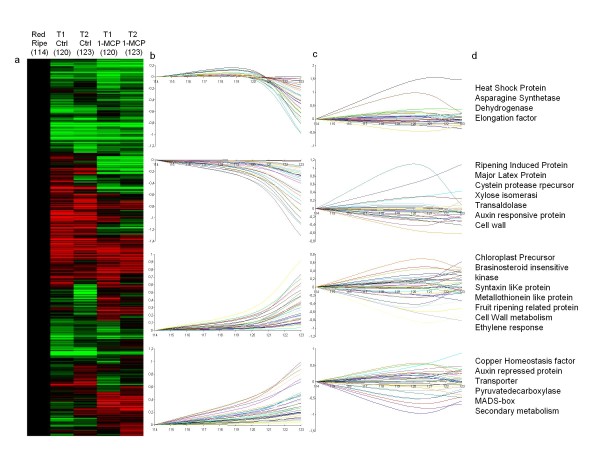
**The effect of 1-MCP on homologous (HOM) transcriptome dynamics during the final stages of apple ripening**. Panel (a) heatmap representing gene expression between 114 DAFB (red ripe) and 123 DAFB (T2 stage). Control samples and samples treated with 1-MCP are shown. Panel (b) shows four distinct expression profiles identified for the final stages of apple ripening (114 DAFB to 123 DAFB). The X axis represents time (DAFB) and the Y axis represents relative expression level after log_2 _transformation. Panel (c) shows the effect of 1-MCP on the expression of genes shown in panel (b). Panel (d) lists the annotation categories for genes shown in (b) and (c). Abbreviations: DAFB, days after full bloom; Ctrl, control samples; 1-MCP, samples treated with 1-Methylcyclopropene.

Transcriptome analysis was performed by hybridizing all samples comprising the experimental design (Figure 1) on both the TOM1 and apple arrays containing 12,899 and 1,608 ESTs, respectively (Additional file 1). The total expression data set, represented by 9,663 filtered and normalized features, was organized into two structured SOTA clusters, comparing functional dynamics between normal fruit ripening and that altered by 1-MCP in the following stages: green (66 DAFB), breaker (90 DAFB), red ripe (114 DAFB), T1_Ctrl/1-MCP _(120 DAFB) and T2_Ctrl/1-MCP _(123 DAFB). To highlight the functional differences between control and 1-MCP treatment, the entire expression regime was re-plotted considering only the last stages of the experimental design (red ripe as the reference point, T1_Ctrl/1-MCP _and T2_Ctrl/1-MCP_) focusing on the regulatory effects resulting from the ethylene response inhibitor treatment (Figure 2a and Additional file 2). Four clusters were isolated from the SOTA tree organization with two down- and two up-regulated profiles during normal ripening (Figure 2b). Within these profiles was the transcriptomic variation caused by 1-MCP (Figure 2c) impacting genes involved in hormone biosynthesis/response, cell wall metabolism, transcription and secondary metabolism (Figure 2d). Similar results in terms of classes of annotated genes were also obtained employing the HOM array. In this case the general profile was represented within a hierarchical clustering (Figure 3a), revealing that the 1-MCP impact on general ripening was similar to that detected with the HET array (Figure 3b, c and 3d).

**Figure 4:**
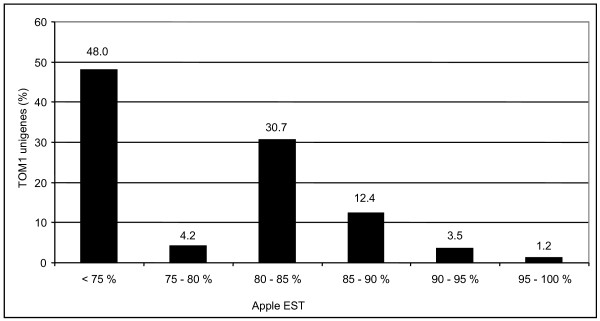
**Nucleotide sequence comparison between the TOM1 sequences and the apple EST collection**. The Y axis indicates the percentage of TOM1 unigene sequences matching with apple unigenes; the X axis shows the % identity for each category.

Gene expression dynamics were analyzed over the climacteric time course using a permutation-based paired-sample t-test performed with BRB-Array Tools, which identified 652 DEGs (Differentially Expressed Genes) in the HET data set (Additional file 3) and 139 DEGs using the HOM array (Additional file 4). *In silico *cross-species nucleotide sequence alignment was performed comparing the total set of 7,352 annotated tomato unigenes comprising TOM1 with the public apple EST collection http://www.rosaceae.org. General similarity analysis was performed via Blastn with a cut-off value of 1 × 10e^-5^. In this analysis, 52% of the genes were considered homologous, as defined by a nucleotide sequence identity greater than 75% (Figure 4). Out of 652 differentially expressed HET array unigenes associated with apple ripening, 430 matched to a corresponding apple sequence with an identity value higher than the 75% considered necessary for designation as "homologous" (Additional file 5). This analysis yielded results similar to what was reported in a prior Solanaceae cross species analysis, where 75% of the available sequences of pepper and eggplant were homologous with those on the tomato array [39]. While apple is more distant from tomato than other members of the Solanaceae, it is noteworthy that the TOM1 array was weighted toward fruit development related genes and these sequences may be more conserved among climacteric fruits. The comparison between tomato and Arabidopsis revealed a lower identity value, further supporting the hypothesis that the TOM1 array might be enriched for conserved fruit-associated genes and thus for using the tomato array as a reference for gene expression studies in fleshy fruits. Apple is a fleshy, climacteric, indehiscent fruit like tomato, while Arabidopsis has non-fleshy, non-climacteric and dehiscent fruit.

It is noteworthy that the difference in array features between the tomato and apple arrays is correspondingly reflected mainly in the number of elements present in each gene category, with 652 DEGs grouped in 24 classes, while the 139 homologous DEGs in apple were grouped in 19 categories - only 20% less (Additional file 6). To highlight the genes differentially impacted by 1-MCP (i.e. those which are ethylene regulated) we compared the profiles of the control samples with their 1-MCP treated counterparts (T1_Ctrl/1-MCP_-T2 _Ctrl/1-MCP_). Based on Pearson correlation (r < 0.25) we observed 7% and 25% differential expression using the HET and HOM arrays, respectively. It is interesting to note that 1-MCP treatment caused up-regulation of a gene set normally repressed in the presence of ethylene (Figure 2a and Additional file 7) which contains members functionally associated with nucleic acid metabolism (RNA binding protein), protein biosynthesis (60S ribosomal protein), signal transduction (Serine/threonine kinase) and gene transcription (bell-like2, MADS-boxGDEF1, WRKY). Using both arrays we identified elements representing genes up-regulated by 1-MCP treatment including IAA7, ribosomial protein, pyruvate decarboxylase, a ripening-related protein of unknown function and a heat shock protein. This negative response to ethylene was also observed in a preliminary 2-D proteomic comparison between T1_Ctrl/1-MCP _(Additional file 8). Specifically, *in silico *proteomic comparisons revealed 590 common peptide spots (57.39%) from the total number of 1,108 and 948 spots for the control and 1-MCP treated, respectively. 1-MCP treatment revealed 358 unique spots, corresponding to 37.8% of the total protein pattern. The difference in the percentage of impacted genes versus proteins likely reflects the fact that the proteomics analysis we performed is likely to over-emphasize the more abundant structural proteins and under-represent the less abundant regulatory peptides suggesting that a greater number of low abundance and putative regulatory proteins may be impacted by 1-MCP. Alternatively (or in addition) this discrepancy may reflect the fact that multiple gene family members may encode proteins that cannot be distinguished via the 2-D analysis. More comprehensive proteomics analysis that results in peptide identification would be required to sort out these or other possibilities.

The number of genes and proteins observed to be impacted by 1-MCP treatment is indeed quite significant especially considering that in this work the 1-MCP treatment occurred at harvest (114 DAFB) when ripening is well underway. In the HOM array we observed a higher rate of genes influenced by 1-MCP, but it must be noted that while the HET array is enriched for fruit-related sequences [27] the HOM array is a fully fruit dedicated array, and thus even more genes than in the HET array would be anticipated to display differential expression during ripening and as a consequence of 1-MCP treatment. It is also interesting that we observed an approximately equal ratio of genes either positively or negatively regulated by ethylene, demonstrating that ethylene in apple has an important and complex impact on ripening physiology, regulating both positively or negatively the expression of various genes. Even more interesting is the fact that a considerable number of ripening-related genes were not affected by the hormone, indicating ethylene-independent ripening mechanisms in climacteric apple fruit. Such genes may be especially interesting as candidates for common regulatory control between climacteric and non-climacteric fruits and thus represent a unique set of genes for further investigation. Ethylene dependent and independent genes have been previously reported in melon [9] where it was suggested that in at least some cases members of the same gene family were regulated under these two distinct ripening control processes [40].

### Transcriptional control of ethylene synthesis, perception and signalling in apple

**Figure 5:**
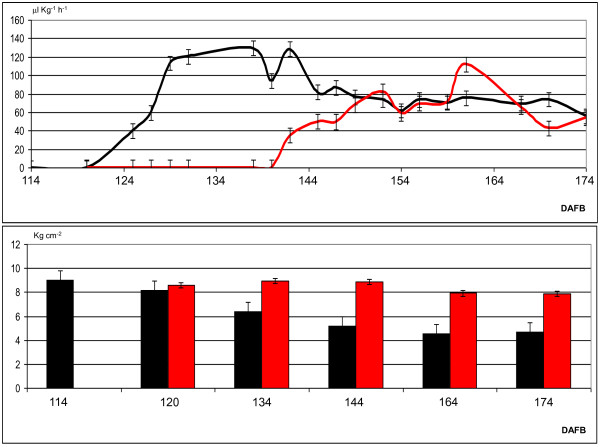
**Ethylene biosynthesis and softening of Mondial Gala apple after 60 days of post-harvest ripening at ambient temperature**. The upper panel shows ethylene evolution during fruit maturation and ripening. The black and red lines indicate ethylene released by control samples and the samples treated with 1-MCP, respectively. Standard error bars are shown. The lower panel shows fruit softening during maturation and ripening. Black and red bars indicate the control samples and the samples treated with 1-MCP, respectively. Standard error bars are shown. Abbreviations: DAFB, days after full bloom.

Mondial Gala fruit development was characterized at three distinct physiological stages: (i) green (66 DAFB, days after full bloom), (ii) breaker (90 DAFB) and (iii) red ripe (114 DAFB). Fruit ripening initiation was defined by the induction of ethylene starting from red ripe (0.65 μlKg^-1^h^-1^) and increasing at T1_Ctrl _(120 DAFB) and T2_Ctrl _(123 DAFB), producing 16.11 μlKg^-1^h^-1 ^and 52.57 μlKg^-1^h^-1 ^of ethylene respectively. Following harvest, 1-MCP application resulted in reduced ethylene synthesis with an ethylene synthesis rate of only 0.66 (T1_1-MCP_) and 0.42 μlKg^-1^h^-1 ^(for T2_1-MCP_), or a reduction in ethylene synthesis of greater than 90% (Figure 1a). To further investigate the efficacy of 1-MCP ethylene repression, we performed a second ethylene assessment extended to 60 days after harvest. At the end of this period the maximum ethylene production in the 1-MCP treated sample was similar to the control (Figure 5), but with a shift of 21 days. Specifically, the control sample produced its maximum (129.53 μlKg^-1^h^-1^) after 26 days following harvest (140 DAFB), while the treated produced 112.12 μlKg^-1^h^-1^, but at day 47 (161 DAFB). These two maximum amounts of ethylene were not statistically different (ANOVA/LSD test, P = 0.05).

**Figure 6:**
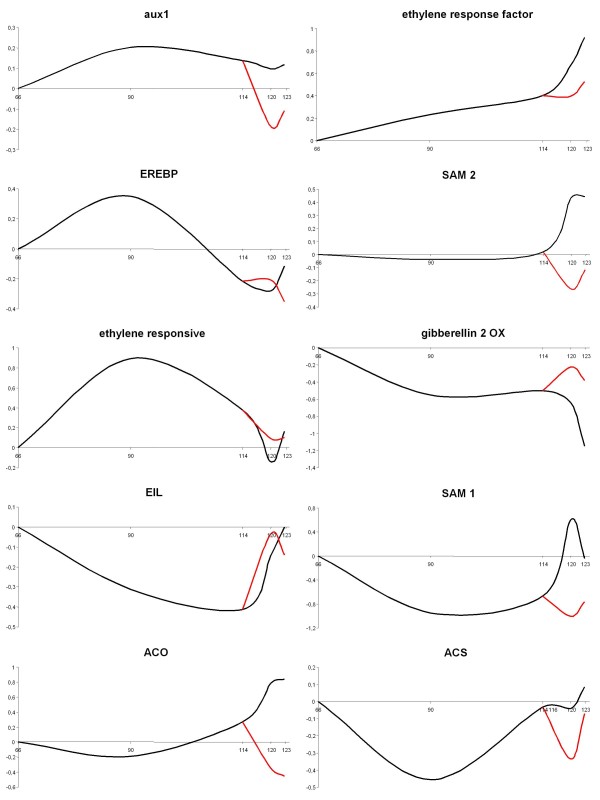
**Expression patterns for genes involved in hormone responses, as determined with the HET array**. The black line indicates the control samples and the red line indicates samples treated with 1-MCP. Abbreviations: 1-MCP, 1-Methylcyclopropene.

HET microarray analysis identified a set of hormone-related DEGs whose expression was differentially coordinate by ethylene. During fruit development, genes involved in the auxin biosynthetic pathway (aux1 protein: SGN-U215673, auxin regulated protein: SGN-U215773, aux protein: SGN-U218763) showed their maximum transcript accumulation at breaker stage (Figure 6 and Additional file 9). A similar elevated expression trend was also observed for genes involved in ethylene response including an ethylene inducible protein (SGN-U214488) and ethylene responsive element (ethylene responsive protease inhibitor I: SGN-U217278, EREBP: SGN-U213917). The TOM1 (HET) data highlighted the ethylene dependent transcriptional control which was additionally supported by 1-MCP application as reflected in down regulation of genes involved in ethylene synthesis and signal transduction which are normally highly expressed during the ethylene burst and include such genes as: SAM 1-2-3 (S-adenosylmethionine synthase: SGN-U212824, SGN-U213593, SGN-U212955), ACS (1-aminocyclopropane-1-carboxylate synthase: SGN-U213523), ACO (1-aminocyclopropane-1-carboxylate oxidase: SGN-U212787), EIL (ethylene insensitive like; SGN-U214759), EREBP (ethylene responsive element binding factors: SGN-U213917) and ERF3 (ethylene responsive factor: SGN-U214815) genes. ERFs are especially interesting as they have documented roles in regulating ethylene responsive genes [41,42], and an ethylene dependent gene expression was confirmed previously through 1-MCP application in apple by Wang et al. [43]. Identification of differentially expressed genes in the ethylene synthesis pathway and cell wall metabolism was also considered validation of the utility of the heterologous array platform. Thus, to additionally confirm HET microarray validity, we assessed the expression of ACO and PG, two genes involved in ethylene biosynthesis and cell wall metabolism (ethylene regulated), respectively, via qPCR in apple. We also interrogated the TED database for tomato *in silico *digital expression profiles (Additional file 10 a and b). In both cases the expression was consistent with that observed in HET and HOM profiling. Specifically, positive regulation at the onset of climacteric ripening and down-regulation upon ethylene inhibition (1-MCP application in apple or analysis of the *Nr *mutation in tomato). We note that in this comparison our main limitation was alignment of the apple and tomato developmental time courses where in tomato the ethylene burst occurs at the breaker stage (42 DAP, days after pollination in cv. Ailsa Craig), while in apple this is a post-harvest phenomena. Nevertheless, in the case of the ACO and PG controls, maximum gene expression was coincident with the hormone burst and declined thereafter in both species and with both the HOM and HET arrays suggesting a degree of reliability in both platforms.

In the gene set negatively correlated with climacteric ethylene production (represented by 21% of DEGs) we identified hormone-related genes associated predominantly with plant growth rather than ethylene with the best hits to members involved in auxin (aux/IAA: SGN-U218763, SGN-U219594) and gibberellin (gibberellin 2-oxidase: SGN-U214290, SGN-U216196) response pathways. Furthermore, in the HOM array we identified seven genes putatively involved in hormone signalling networks. Three were homologous to regulatory genes of the auxin pathway, while the other four were involved in ethylene biosynthesis and signalling: ACO, ethylene receptor and Md-ETR genes (Additional file 11). The expression of both ACO and Md-ETR was ethylene dependent, as both were up-regulated during normal ripening and repressed by 1-MCP.

### Transcriptional control of fruit softening in apple

To establish ties between gene expression pattern and fruit physiology, we analyzed a number of ripening parameters in the same fruit used for expression profiling including measurement of fruit firmness (Figure 1b). Firmness in T1 fruit was 7.5 Kg cm^-2 ^for the control and 8.1 Kg cm^-2 ^for the 1-MCP treated samples and this difference increased in fruit at the T2 stage, with 6 Kg cm^-2 ^for T2_Ctrl _and 8.3 Kg cm^-2 ^for T2_1-MCP, _respectively. 1-MCP treated samples lost only 1.2 Kg cm^-2 ^after 60 days of post-harvest ripening, compared to the 4.4 Kg cm^-2 ^lost by the control (Figure 5).

**Figure 7:**
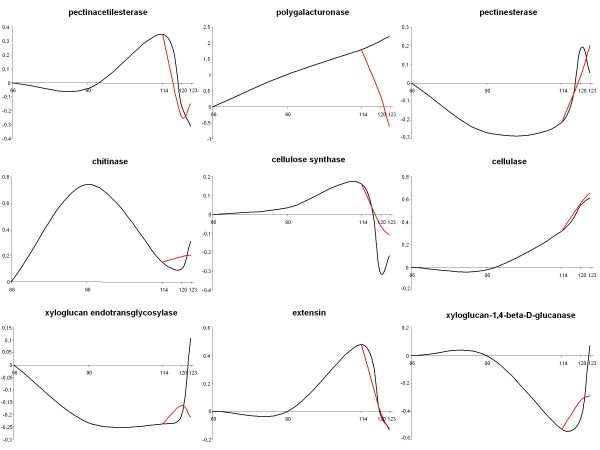
**Expression patterns for genes involved in cell wall metabolism, as determined with the HET array**. The black line indicates the control samples and the red line indicates samples treated with 1-MCP. Abbreviations: 1-MCP, 1-Methylcyclopropene.

Using the HET array we identified 27 DEGs putatively involved in cell wall metabolism (Additional file 3). We observed two general trends of gene expression related to cell wall enzymes (Figure 7 and Additional file 9b). The first trend comprise a set of genes whose maximum expression occurs in the breaker/red ripe stages and then decreases during the post-harvest period, such as pectin acetylesterase (SGN-U217232), cellulose synthase (SGN-U221500), chitinase (SGN-U217904) and extensin (SGN-U214487). The second category is characterized by genes whose maximum transcript abundance is observed at the end of the time course, coincident with the ethylene burst in apple. Transcripts of xyloglucan endotransglycosylases (SGN-U215860), xyloglucan endo 1-4 glucanase (SGN-U217975) and polygalacturonase (SGN-U213213) fall into this category. Our data regarding cell wall gene expression profiling was consistent with the results of others [44-46] reporting both early and late fruit development enzymatic actions associated with fruit softening. Among the genes involved in later stage cell wall metabolism, polygalacturonase, xyloglucan endotransglycosylases and xyloglucan endo 1-4 glucanase, in particular, showed down regulation after 1-MCP treatment, confirming the importance of ethylene and cell wall metabolizing enzymes in fruit softening control. In the HOM array seven cell wall unigenes demonstrated differential expression (Additional file 12) including pectin acetylesterase precursor, endoxyloglucan transferase, xylose isomerase and a polygalacturan gene. According to the HET profile, polygalacturonase found on the HOM array showed an ethylene dependent profile as well, with a maximum release at T2_Ctrl_, and strong down-regulation following 1-MCP treatment.

### Transcription factor gene expression in fruit maturation and ripening

Fruit development and ripening are highly coordinated by an emerging set of transcription factors which have been defined largely in tomato though shown to have counterparts in other climacteric and non-climacteric species [47-51]. Using TOM1, 11.5% of the differentially expressed genes during fruit development and ripening were annotated as putative transcription factors, and 40% of these were up regulated in the preclimacteric phase (between green and red ripe), while the majority were highly expressed at the onset of ripening (from red ripe to the T2 stage), as typical in tomato [3]. Within this category, the most common gene families were MYB, AP2 domain, bZIP, MADS-box, bHLH and WIZZ, a set of transcription factors which are generally the most abundant in all eukaryote genomes sequenced to date [52].

**Figure 8:**
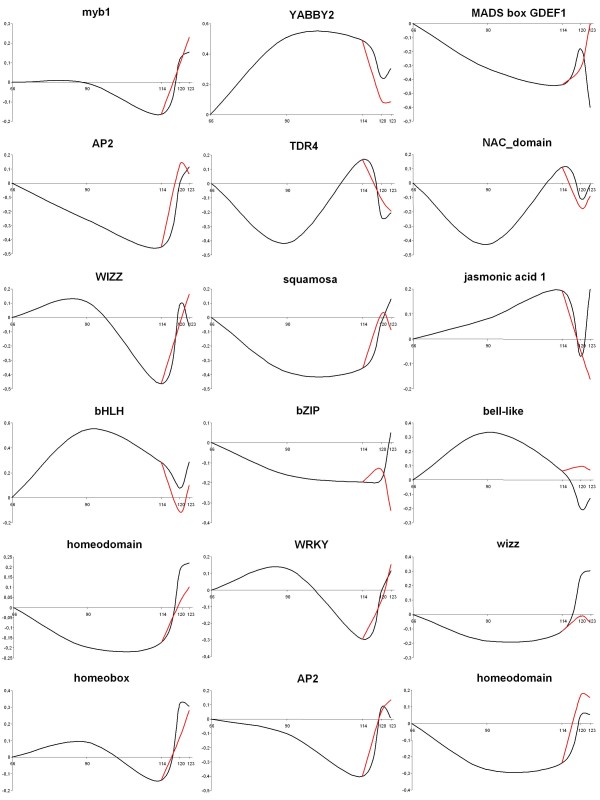
**Expression patterns for genes encoding transcription factors, as determined with the HET array**. The black line indicates the control samples and the red line indicates samples treated with 1-MCP. Abbreviations: 1-MCP, 1-Methylcyclopropene.

1-MCP application affected the expression of 13% of these transcription factor genes (Additional file 9c). Unigenes belonging to the bZIP (SGN-U214146) group and WIZZ (SGN-U213245) were down regulated by 1-MCP treatment (ethylene dependent), while other elements including MADS-box GDEF1 (SGN-U215918), MYB TMH27 (SGN-U215971) and AP2 (SGN-U218041) genes were stimulated by this treatment, suggesting negative regulation by ethylene in a subset of putative ripening regulators (Figure 8). Five AP2 members were identified in the HET array, both developmentally and ripening regulated, in agreement with observations in peach [18].

Transcription factors that showed the highest change in mRNA abundance between the breaker and red ripe stages included bell-like (SGN-U214635), CCR4 (SGN-U213840), jasmonic acid 1 (SGN-U214021), Pti5 (SGN-U217388), YABBY2 (SGN-U213463), MYB (SGN-U215971, SGN-U215897), SET domain (SGN-U225149), ZPT2 (SGN-U213138), bZIP (SGN-U220645, SGN-U220052), bHLH (SGN-U223789) and AP2 (SGN-U219020). In addition to those induced during fruit development, others showed a unique profile of being primarily specific to ripening/ethylene expression such as homeodomain (SGN-U213729), bZIP (SGN-U214147), Dof-zinc (SGN-U218870), TINY (SGN-U224037), WRKY (SGN-U212725, SGN-U245688, SGN-U214107), WIZZ (SGN-U213245) and NAM (SGN-U220043). Transcription factors belonging to AP2, bHLH, bZIP, homeobox, MADS, MYB and the squamosa families showed complex and unique fruit development and ripening regulation, with different members of these families induced in both periods. The role of bZIP in fruit ripening has been documented in other species such as watermelon [53], tomato [54] and strawberry [55]. In climacteric fruits the role of MADS-box genes has been previously confirmed in tomato via demonstration of the necessity of LeMADS-RIN for ripening [49] and of MdPI in apple seed and fruit development [51]. The relevance of this family in these physiological processes was also supported by interaction studies where 5 MADS-box elements including TDR4 were shown to interact *in vitro *with LeMADS-RIN [1,56]. In the current HOM apple data set only two transcription factors have been identified (Additional file 13): a leucine rich repeats (LRR) protein, and a MADS box gene further supporting the value of the HET data developed here. Both genes were positively regulated by ethylene. 1-MCP application caused dramatic repression of both genes further supporting the role of ethylene in the regulation of these genes.

To have a better picture of fruit transcriptomics, the entire HOM transcription profile was organized in a functional dendrogram which revealed two main clusters (Additional file 14). The 1-MCP treated samples clustered in a group closer to the earlier pre-ripening developmental stages (green and breaker). In fact, within this DEG group were identified elements typical of organs in development, involved in energy biosynthesis, nucleic acid metabolism and transcriptional control. By this functional correlation it is evident that during fruit development and ripening the change between the breaker and ripening stage are determined in large part by differential gene expression.

Together with ethylene receptors, transcription factors represent key developmental timing systems [57]. A delayed ethylene burst due to 1-MCP could induce the plant to activate compensatory regulatory machinery in order to re-establish normal ripening physiology. Extending the fruit post-harvest observation up to 60 days after harvest we have in fact observed that the sample treated with 1-MCP reached almost the same maximum amount of ethylene suggesting such feed back control mechanisms operate during ripening.

### Conserved expression dynamics in apple and tomato fruit

One of our primary objectives was to add to the reservoir of ripening knowledge by identifying genes co-associated with ripening and ethylene response in both tomato and apple. Toward this end we compared the 652 genes differentially expressed in this work with the 869 tomato homologous genes reported by Alba et al. [28]. Comparison of these two data sets (detected with the same platform: TOM1), identified 157 genes common to both data sets. Of these, 108 genes had apple orthologous sequence counterparts with higher than 75% identity, representing a putative gene set of homologs involved in the ripening of both species (Table 1 and Additional file 15). A similar number of genes (102) differentially expressed over the course of fruit development of both apple and tomato was reported by Janssen et al. [23], however, only 20% of these genes were identified in this study so that a new and larger collection of highly homologous ripening-associated genes is available from tomato and apple. Such genes represent a foundation from which candidate conserved genes among other fruit species can be mined. The major differences with the Janssen report were likely due to the differences between experimental designs. In their case the samples collected where more representative of the pre-ripening fruit development and initial maturation, while this work focused on the later development and post-harvest ripening. As such this work focuses on the climacteric stage and the corresponding link with the ethylene production was confirmed through the finding of twelve microarray elements representing genes involved in hormone production and response (SAM, ACS and ACO). In Janssen et al. [23] six genes were defined as ethylene related and all reflected SAM synthase genes, one of the early enzymes in the ethylene biosynthetic pathway. In Janssen et al. [23] five genes were found related to cell wall metabolism in common with tomato, of which two are α-expansin, here showed to be more active during the initial fruit softening stage. Our work identified 6 cell wall sequences in common with tomato though more related to later stages of fruit softening (e.g. polygalacturonase, xyloglucan endo-1,4 glucanase). The combination of these two data sets improves the common genomic comparison between apple and tomato, expanding the number of possible genes commonly active in both (and presumably other) species from early development through post-harvest ripening.

**Table 1:** Apple and Tomato Ripening Genes

Tomato_ID	Apple_ID	Gene_annotation
		*cell wall; carbohydrate metabolism; cell wall degredation; fruit softening*
SGN-U212775	CN29021	pectate lyase [Malus x domestica]
SGN-U217975	CN25519	xyloglucan endo-1,4-beta-D-glucanase (EC 3.2.1.-) precursor (clone tXET-B2) - tomato
SGN-U213213	CN14797	Polygalacturonase 2A precursor (PG-2A) (Pectinase)
SGN-U213444	CN27677	xyloglucan endotransglycosylase (XTR4), putative [Arabidopsis thaliana]
		
		*defense responses; disease resistance; pathogenesis; wound induced*
SGN-U219296	CX022916	ELI3 [Lycopersicon esculentum]
		
		*hormone responses; auxin responses; IAA responses*
SGN-U215673	CN7981	axi 1 protein from Nicotiana tabacum -related [Arabidopsis thaliana]
		
		*hormone responses; ethylene responses*
SGN-U214488	CN24915	ETHYLENE-INDUCIBLE PROTEIN HEVER
SGN-U212804	CN445336	ACC oxidase homolog (Protein E8)
SGN-U214815	CN28691	ethylene response factor 3 [Lycopersicon esculentum]
		
		*hormone responses; ethylene biosynthesis*
SGN-U212786	CN309	ACC oxidase, 1-aminocyclopropane-1-carboxylate oxidase 1 (ACC oxidase 1)
SGN-U212787	CN27	ACC oxidase, 1-aminocyclopropane-1-carboxylate oxidase 1 (ACC oxidase 1)
SGN-U214919	CN309	ACC oxidase, 1-aminocyclopropane-1-carboxylate oxidase [Solanum tuberosum]
SGN-U216896	U73815	ACC synthase, 1-aminocyclopropane-1-carboxylate synthase 2
SGN-U212824	CN1498	S-adenosylmethionine synthetase 1
SGN-U212955	CN14011	S-adenosylmethionine synthetase 3
SGN-U213593	CN1498	S-adenosylmethionine synthetase 2
		
		*ripening-related*
SGN-U213072	CN5470	ripening-related protein [Vitis vinifera]
		
		*transcription factor*
SGN-U213245	CO052409	WIZZ [Nicotiana tabacum]
SGN-U213317	CN791	transcription factor BTF3 (RNA polymerase B transcription factor 3)
SGN-U213318	CN791	transcription factor BTF3 (RNA polymerase B transcription factor 3)
SGN-U213318	CN791	transcription factor BTF3 (RNA polymerase B transcription factor 3)
SGN-U213659	CN27467	TDR4 transcription factor [Lycopersicon esculentum]
SGN-U213840	CN26889	CCR4-associated factor -related [Arabidopsis thaliana]
SGN-U215425	CN8887	bZIP transcription factor BZI-4 [Nicotiana tabacum]
SGN-U215688	CN25810	WRKY family transcription factor [Arabidopsis thaliana]
SGN-U215971	CN1890	myb-related protein TMH27 - tomato
SGN-U217991	CN495178	AP2 domain transcription factor, putative [Arabidopsis thaliana]
SGN-U219020	CN495178	AP2 domain transcription factor, putative [Arabidopsis thaliana]

Twenty-eight genes that are differentially expressed in the ripening of apple and tomato (Alba et al., 2005 and Fei et al., 2006), specifically involved in ethylene biosynthesis and response, cell wall metabolism and transcription factors.

The heterologous expression profiles of the 108 common genes for apple were compared with tomato *in silico *digital expression data retrieved from the TED database http://ted.bti.cornell.edu/. Pearson correlation confirmed that over the course of normal climacteric ripening almost 70% of the genes identified by array analysis were consistent with the digital expression results, again providing validation for the HET array assay in apple and suggesting equally useful results could be recovered from other fruit species to expand the comparative fruit genomics base.

Analyzing the digital expression profile of the common gene set using the WT vs. *Nr *tomato comparison, we observed differential expression of 35.7% of genes, consistent with what was reported by Alba et al. [28]. In apple, for the same gene set identified with the same array, 1-MCP resulted in differential expression of 30% of these genes, consistent with the tomato results and suggesting that these elements might have a common regulatory role in ripening control of both tomato and apple.

## Conclusion

In this work we presented a heterologous approach to investigate the transcriptome of apple ripening and common genes with tomato that may serve as a base collection of candidates for conserved ripening regulation among diverse fruit species. The HET tomato array was used with the principal goal of identifying candidate genes related to fruit development and ripening that could then be related to previously characterized tomato ripening genes. Gene validation was partially gained by parallel hybridization of the same set of samples with the HOM array (which is apple-specific but of limited size).

Heterologous microarray platforms could provide a useful alternative to explore transcriptome dynamics in the absence of a whole genome array and EST data. Here we have demonstrated the use of a tomato array to identify putative apple sequences which are associated with ripening and thus may be targets for further fruit ripening and comparative genomics studies. At present to the scientific community is presented a growing number of advanced next generation sequencing technologies (NGST) that provide a viable alternative to microarray analysis [58]. Despite their great potential, these new technologies still present some bias mainly related to technical features of the outputs [59] and the need for strong bioinformatics support to exploit these data. With the recent availability of the apple genome [60] and the impending release of the tomato genome, these NGST will certainly open new possibilities to target gene expression with high fidelity such as the repertoire of candidate ripening and evolutionarily conserved fruit genes that can be further expanded between tomato and apple and extended to additional important fruit crop species.

## Authors' contributions

FC designed the experiment, collected the samples, performed the microarray hybridization, analyzed the data and wrote the manuscript. RA supported the microarray hybridization, contributed to the data analysis and revised the manuscript. HS provided the apple array. VS and LG supported the interpretation and annotation of the homologous array. SSerra and SM performed the 2-D proteomic assay. SSansavini supported the sample collection. GC supported the 1-MCP treatment and gas-chromatography analysis for ethylene. ZF performed the bioinformatics analysis. JG contributed to the experiment design, provided important advise during the work and improved the manuscript.

All authors read and approved the final manuscript.

## Supplementary Material

Additional file 1 **Comparison between the hybridization of the tomato array (a) and the apple array (b) with apple cDNA**. Images (c) and (d) represent the sub-grid magnification for both arrays. Click here for file

Additional file 2 **Comparative dynamics between the control (a) and 1-MCP treated (b) sample. The 3D plot refers to the up regulated profile**. In the boxes are highlighted specific genes of the functional profile. Click here for file

Additional file 3 **DE genes in apple identified using the heterologous (HET) array TOM1**. Click here for file

Additional file 4 **DE genes in apple identified using the homologous (HOM) apple array**. Click here for file

Additional file 5 **TOM1 unigenes homologous to the apple EST dataset collection**. Homology is defined by 75% identity. Click here for file

Additional file 6 **GO annotations for DE genes identified with the HET (a) and HOM (b) arrays**. Tables include GO annotations, the number of unigenes identified, and the relative percentage of unigenes identified. Click here for file

Additional file 7 **Comparative dynamics between the control sample (a) and the 1-MCP treated sample (b)**. The profiles refer to down regulation. In this particular case a negative regulation is reflected into an up regulation in the positive part of the plot (framed box). Click here for file

Additional file 8 **2D proteomic comparison profile carried out using T1_Ctrl _and T1_1-MCP _samples**. Each synthetic gel has been obtained from 3 gels per sample. Colored squares represent the anchors used to facilitate the comparison. Data at the bottom of the figure summarize spot numbers and the relative matching values. Click here for file

Additional file 9 **Hierarchical clustering of gene expression patterns identified with the HET array**. Three functional categories are shown: hormone pathways (a), transcription factors (b) and cell wall enzymes (c). The three clusters show functional dynamics of late ripening and comparison with 1-MCP. Samples are coded as RR for red ripe: C1 and C2 for T1 and T2 Control respectively; M1 and M2 for T1 and T2 1-MCP treated respectively. Click here for file

Additional file 10 **Expression profiles for ACO and PG in apple and tomato**. (a) shows expression profiles in developing apple fruit, as determined by qPCR. (b) shows digital expression profiles in tomato fruit, as retrieved from the TED database. Data for ACO and PG are shown in red and black, respectively. The solid line indicates the control samples and the dashed line indicates samples treated with 1-MCP. Abbreviations: DAFB, days after full bloom; DAP, days after pollination; 1-MCP, 1-Methylcyclopropene. Click here for file

Additional file 11 **Expression patterns for genes involved in hormone responses, as determined with the HOM array**. The black line indicates the control samples and the red line indicates samples treated with 1-MCP. Abbreviations: 1-MCP, 1-Methylcyclopropene. Click here for file

Additional file 12 **Expression patterns for genes involved in cell wall metabolism, as determined with the HOM array**. The black line indicates the control samples and the red line indicates samples treated with 1-MCP. Abbreviations: 1-MCP, 1-Methylcyclopropene. Click here for file

Additional file 13 **Expression patterns for genes encoding transcription factors, as determined with the HOM array**. The black line indicates the control samples and the red line indicates samples treated with 1-MCP. Abbreviations: 1-MCP, 1-Methylcyclopropene. Click here for file

Additional file 14 **Expression clustering dendrogram with centered correlation and average linkage**. The cluster was produced using HOM array data and shows the expression profile similarity among samples. Click here for file

Additional file 15 **Eighty genes (complementary to **table 1**) expressed during the ripening of both apple and tomato**. Click here for file
