# Supplementary figures and images for: Use of homologous and heterologous gene expression profiling tools to characterize transcription dynamics during apple fruit maturation and ripening
Source: BMC Plant Biol. 2010 Oct 25;10:229. doi: 10.1186/1471-2229-10-229 (PMC3095317; doi:10.1186/1471-2229-10-229)

## Slide 1
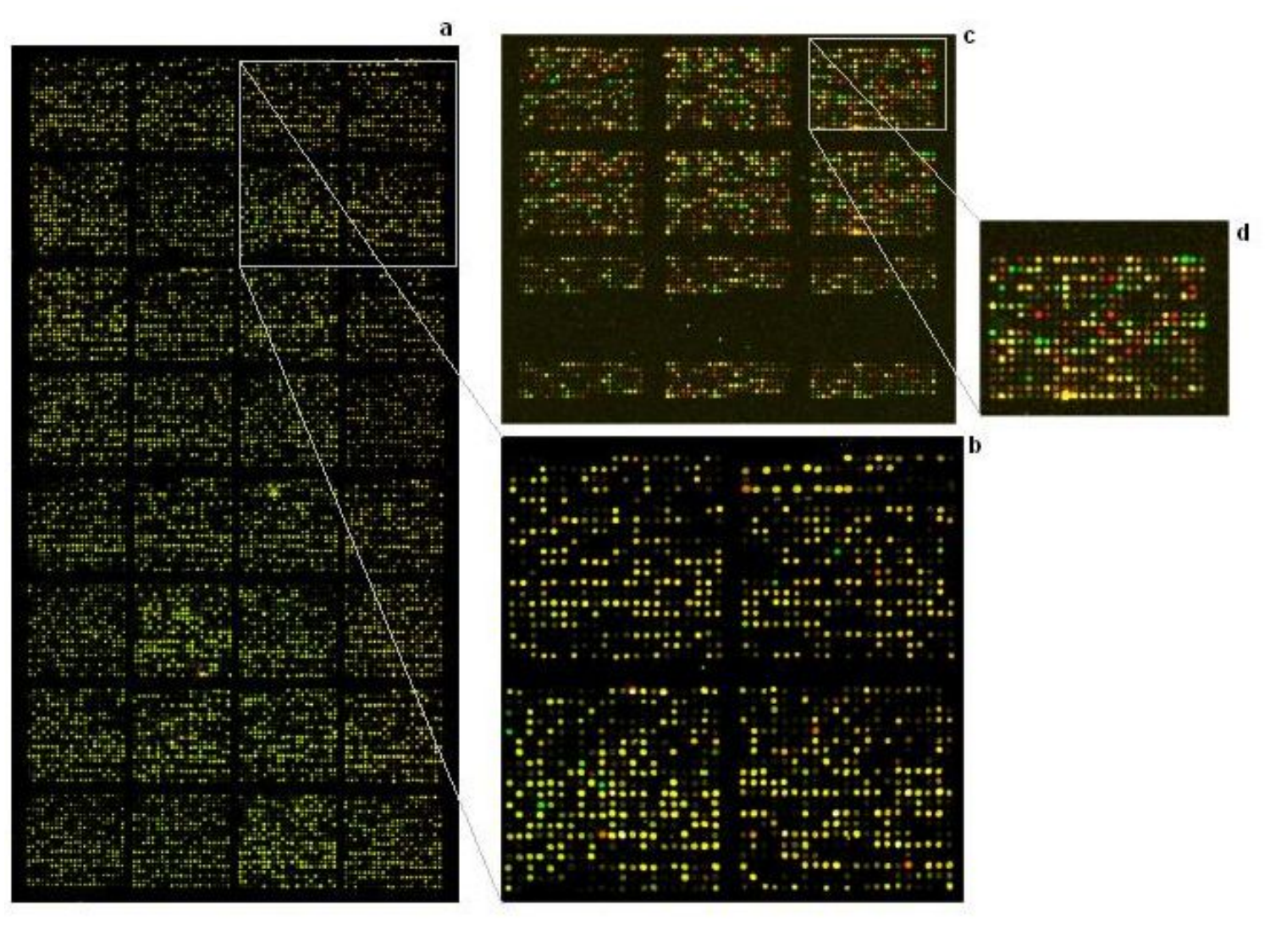

Supplement: Additional file 1 — Comparison between the hybridization of the tomato array (a) and the apple array (b) with apple cDNA. Images (c) and (d) represent the sub-grid magnification for both arrays. [file 1471-2229-10-229-S1.PPT]

## Slide 1
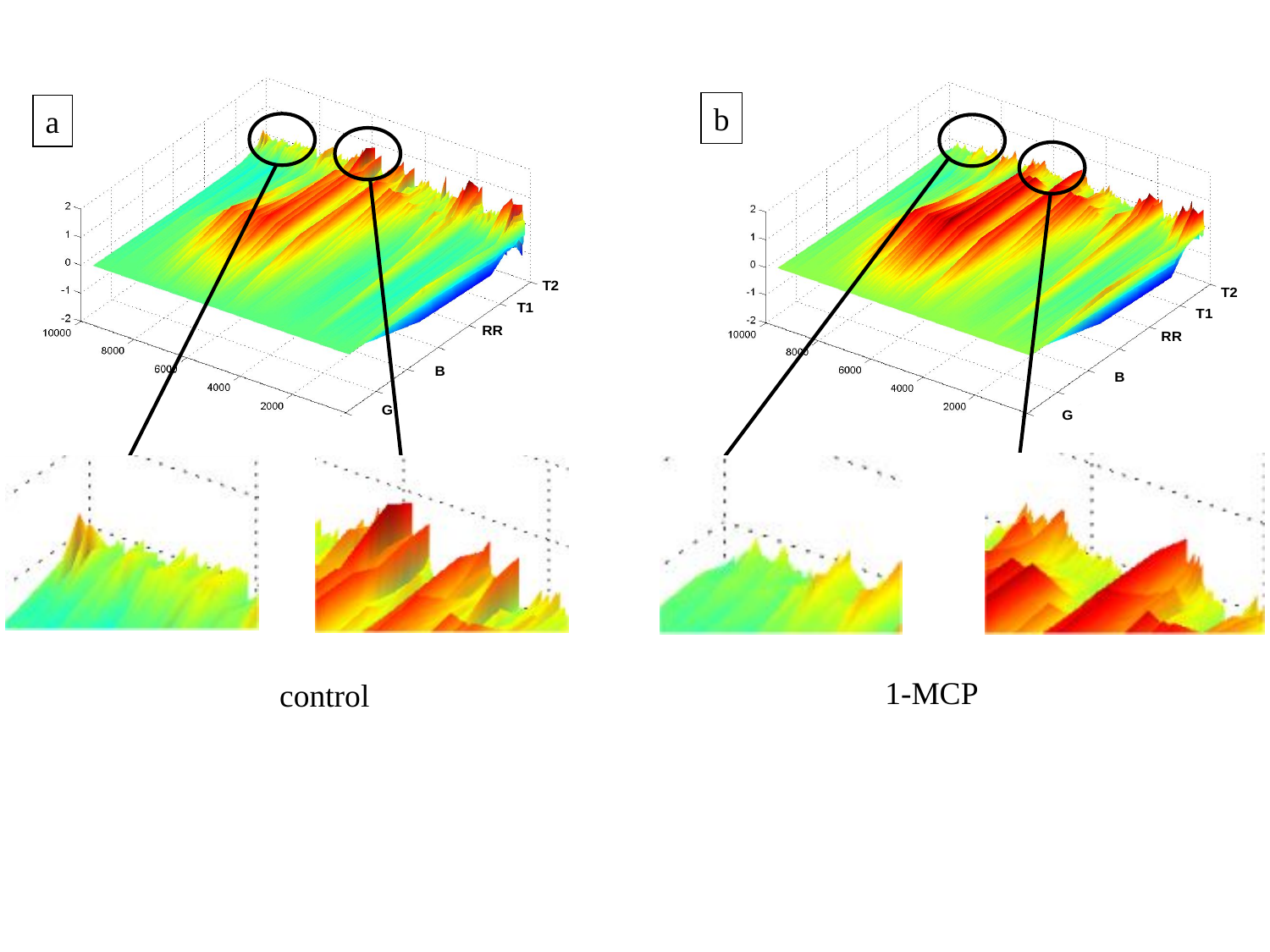

b
a
1-MCP
control

Supplement: Additional file 2 — Comparative dynamics between the control (a) and 1-MCP treated (b) sample. The 3D plot refers to the up regulated profile. In the boxes are highlighted specific genes of the functional profile. [file 1471-2229-10-229-S2.PPT]

## Slide 1
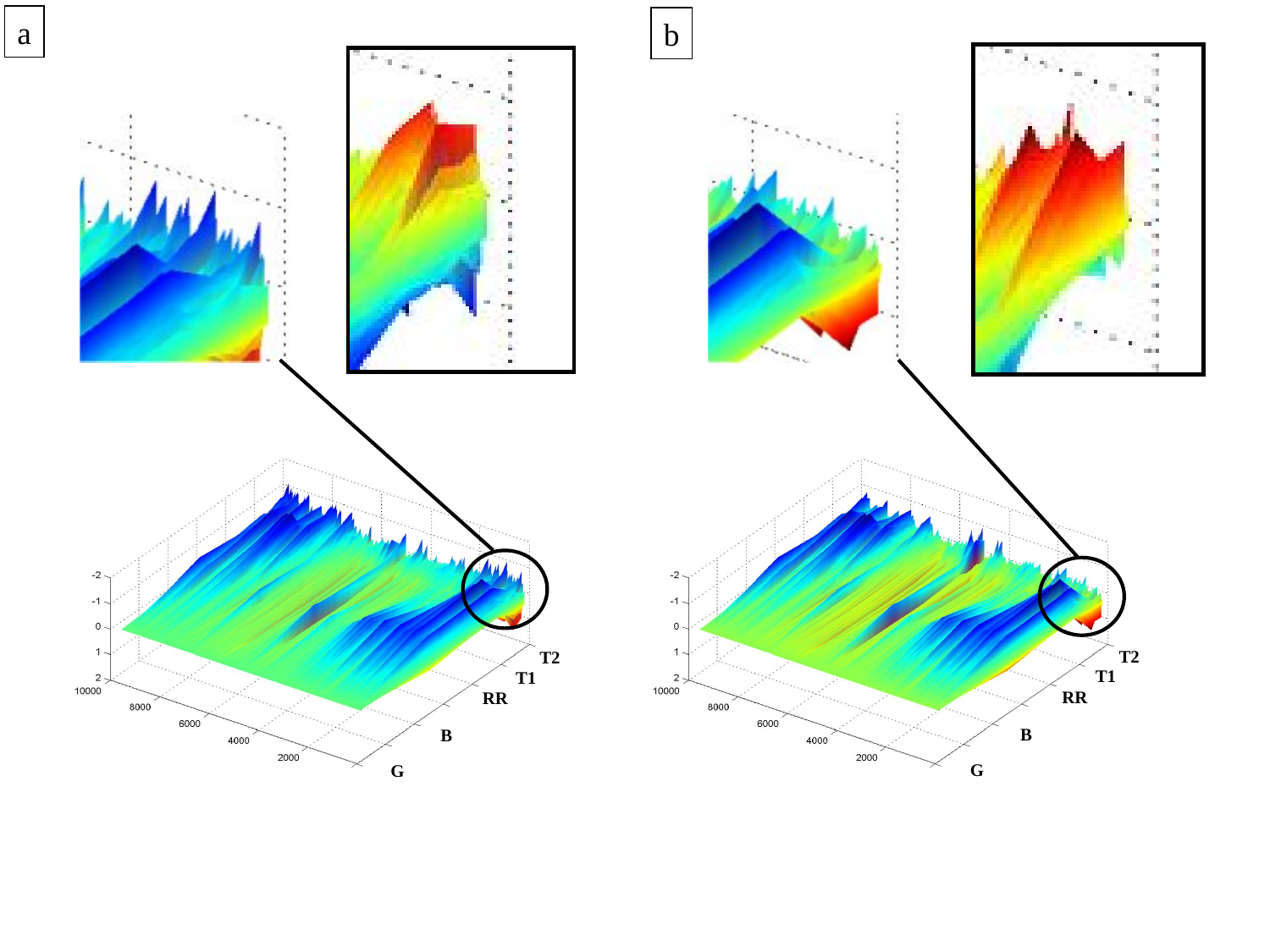

a
b
T2
T2
T1
T1
RR
RR
B
B
G
G

Supplement: Additional file 7 — Comparative dynamics between the control sample (a) and the 1-MCP treated sample (b). The profiles refer to down regulation. In this particular case a negative regulation is reflected into an up regulation in the positive part of the plot (framed box). [file 1471-2229-10-229-S7.PPT]

## Slide 1
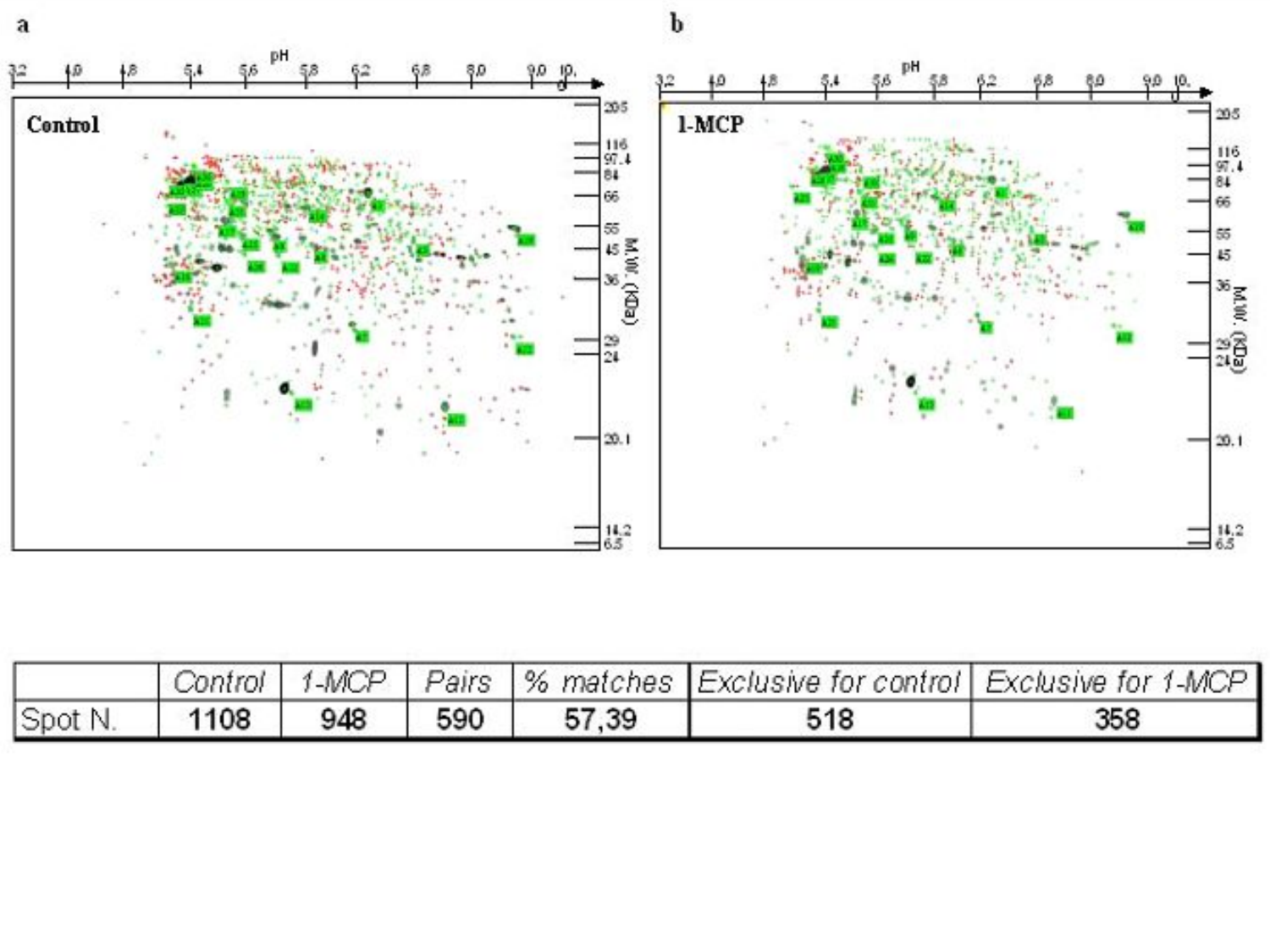

Supplement: Additional file 8 — 2D proteomic comparison profile carried out using T1Ctrl and T11-MCP samples. Each synthetic gel has been obtained from 3 gels per sample. Colored squares represent the anchors used to facilitate the comparison. Data at the bottom of the figure summarize spot numbers and the relative matching values. [file 1471-2229-10-229-S8.PPT]

## Slide 1
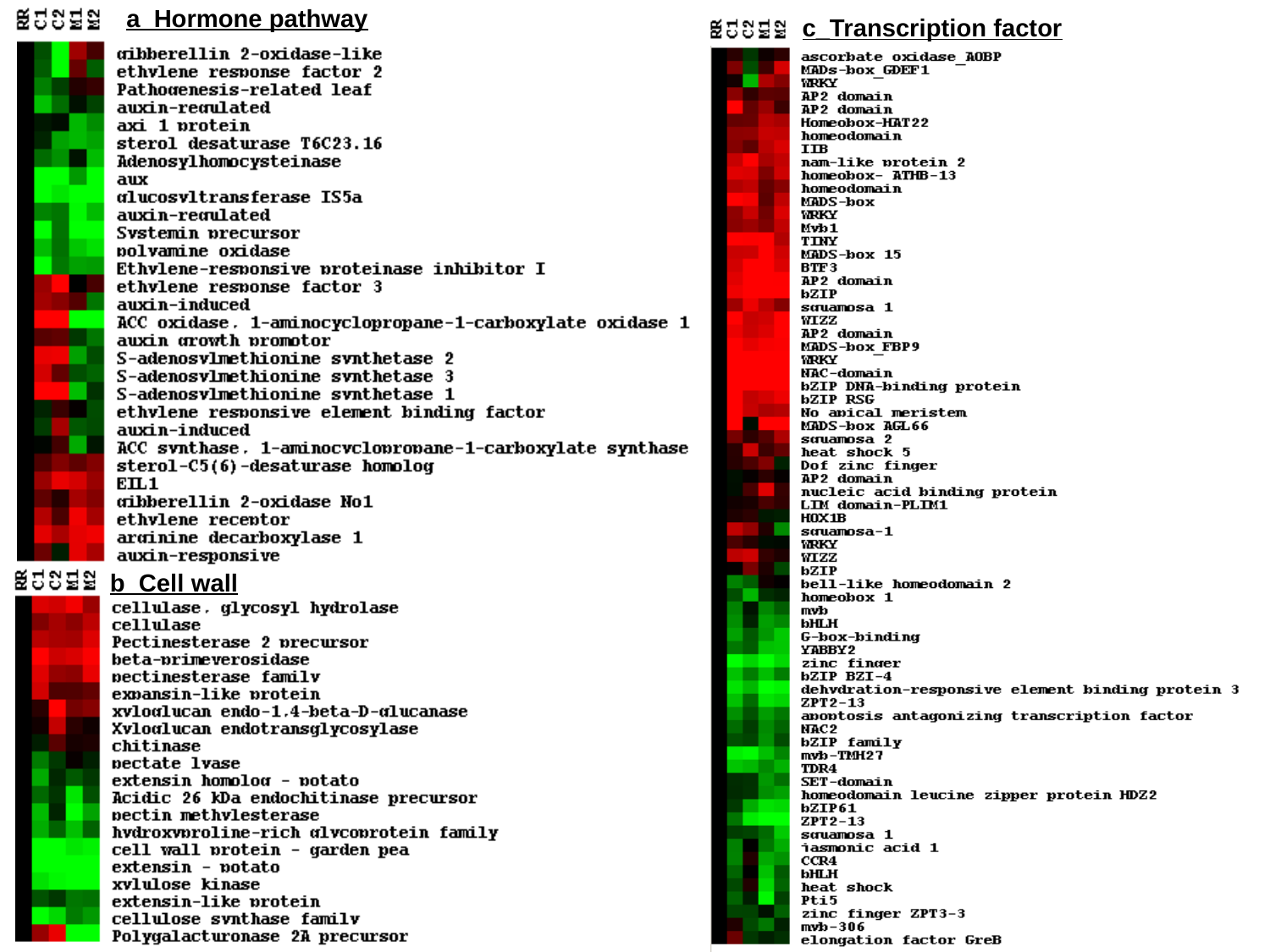

a_Hormone pathway
c_Transcription factor
b_Cell wall

Supplement: Additional file 9 — Hierarchical clustering of gene expression patterns identified with the HET array. Three functional categories are shown: hormone pathways (a), transcription factors (b) and cell wall enzymes (c). The three clusters show functional dynamics of late ripening and comparison with 1-MCP. Samples are coded as RR for red ripe: C1 and C2 for T1 and T2 Control respectively; M1 and M2 for T1 and T2 1-MCP treated respectively. [file 1471-2229-10-229-S9.PPT]

## Slide 1
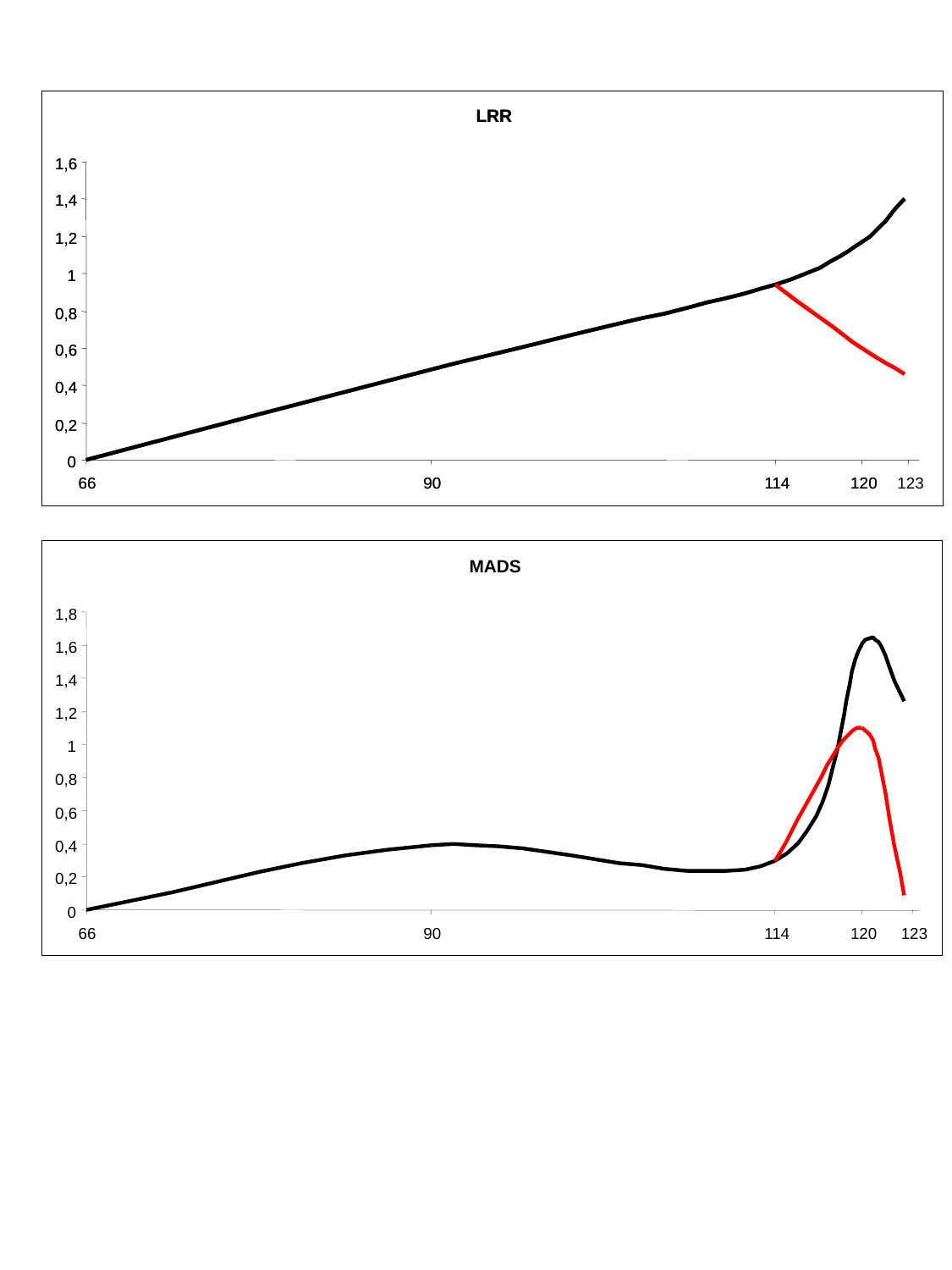

LRR
LRR
1,6
1,6
1,4
1,4
1,2
1,2
1
1
0,8
0,8
0,6
0,6
0,4
0,4
0,2
0,2
0
0
123
66
66
90
90
114
114
120
120
MADS
MADS
1,8
1,8
1,6
1,6
1,4
1,4
1,2
1,2
1
1
0,8
0,8
0,6
0,6
0,4
0,4
0,2
0,2
0
0
66
66
90
90
114
114
120
120
123
123

Supplement: Additional file 13 — Expression patterns for genes encoding transcription factors, as determined with the HOM array. The black line indicates the control samples and the red line indicates samples treated with 1-MCP. Abbreviations: 1-MCP, 1-Methylcyclopropene. [file 1471-2229-10-229-S13.PPT]

## Slide 1
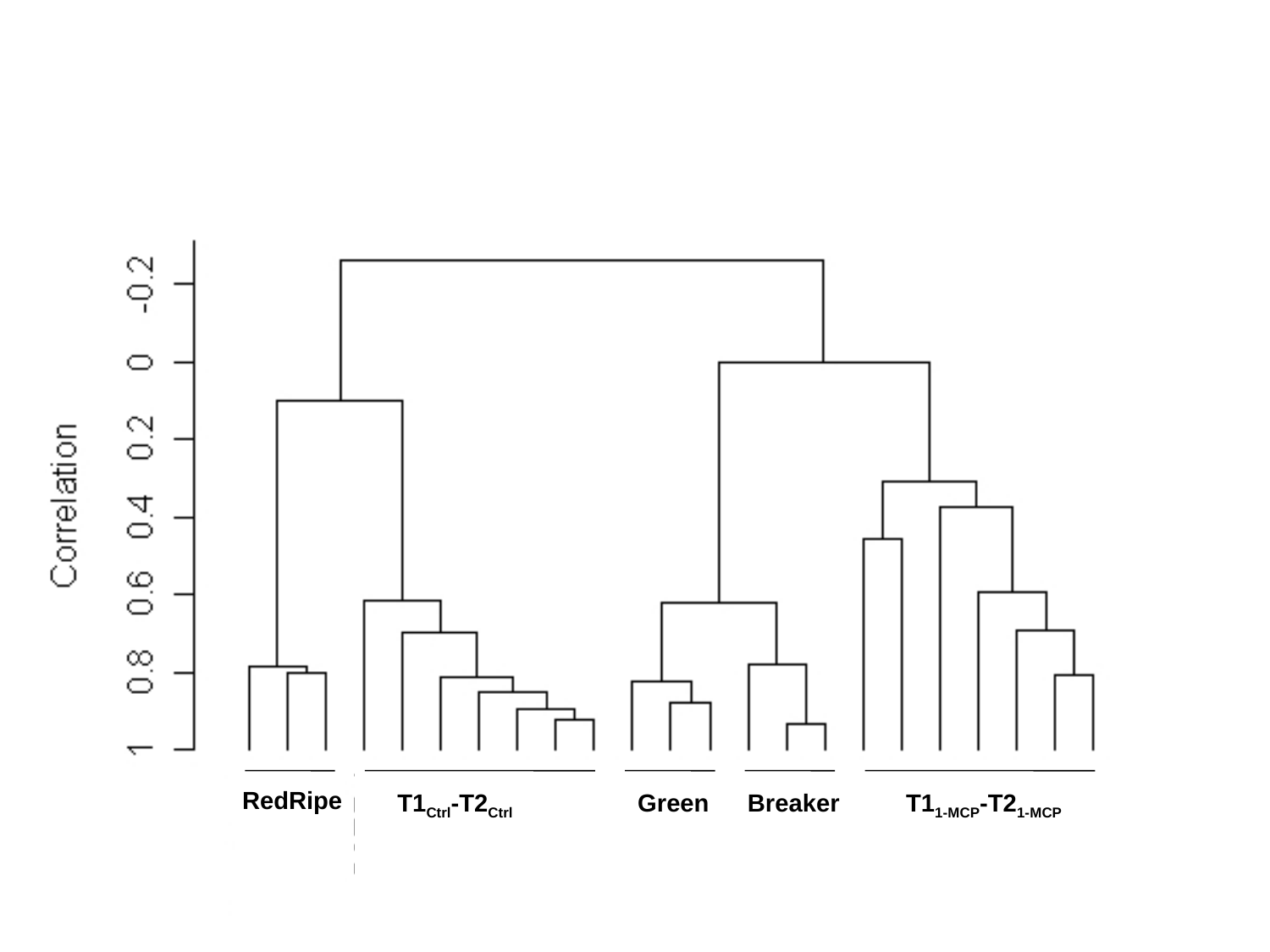

RedRipe
T1Ctrl-T2Ctrl
Green
Breaker
T11-MCP-T21-MCP

Supplement: Additional file 14 — Expression clustering dendrogram with centered correlation and average linkage. The cluster was produced using HOM array data and shows the expression profile similarity among samples. [file 1471-2229-10-229-S14.PPT]
