# Supplementary material for: Use of homologous and heterologous gene expression profiling tools to characterize transcription dynamics during apple fruit maturation and ripening
Source: BMC Plant Biol. 2010 Oct 25;10:229. doi: 10.1186/1471-2229-10-229 (PMC3095317; doi:10.1186/1471-2229-10-229)
Supplement: Additional file 10 — Expression profiles for ACO and PG in apple and tomato. (a) shows expression profiles in developing apple fruit, as determined by qPCR. (b) shows digital expression profiles in tomato fruit, as retrieved from the TED database. Data for ACO and PG are shown in red and black, respectively. The solid line indicates the control samples and the dashed line indicates samples treated with 1-MCP. Abbreviations: DAFB, days after full bloom; DAP, days after pollination; 1-MCP, 1-Methylcyclopropene. [file 1471-2229-10-229-S10.PPT]

## Slide 1
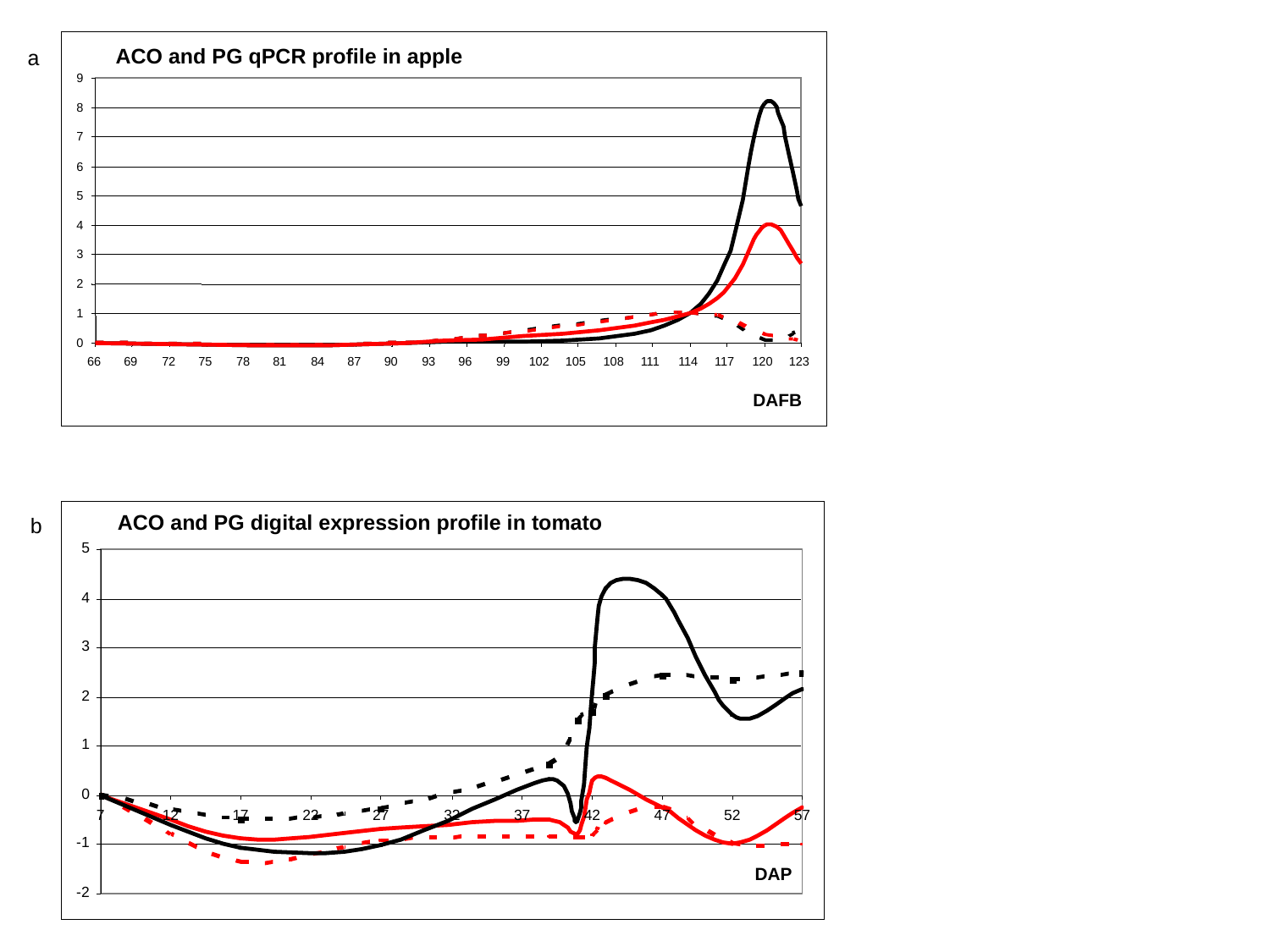

ACO and PG qPCR profile in apple
a
9
8
7
6
5
4
3
2
1
0
66
69
72
75
78
81
84
87
90
93
96
99
102
105
108
111
114
117
120
123
DAFB
ACO and PG digital expression profile in tomato
b
DAP
