# Supplementary material for: Use of homologous and heterologous gene expression profiling tools to characterize transcription dynamics during apple fruit maturation and ripening
Source: BMC Plant Biol. 2010 Oct 25;10:229. doi: 10.1186/1471-2229-10-229 (PMC3095317; doi:10.1186/1471-2229-10-229)
Supplement: Additional file 11 — Expression patterns for genes involved in hormone responses, as determined with the HOM array. The black line indicates the control samples and the red line indicates samples treated with 1-MCP. Abbreviations: 1-MCP, 1-Methylcyclopropene. [file 1471-2229-10-229-S11.PPT]

## Slide 1
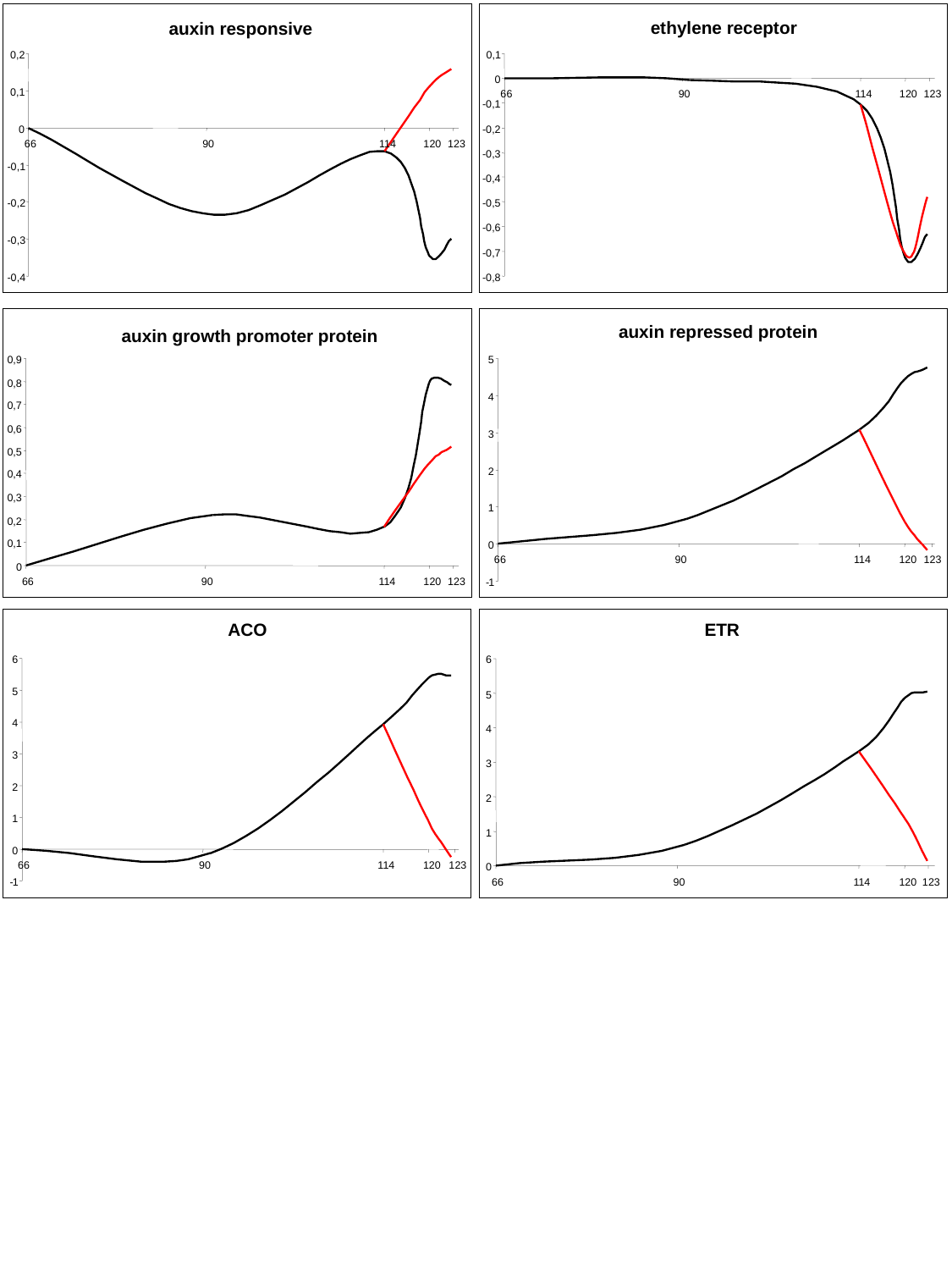

ethylene receptor
auxin responsive
auxin
responsive
ethylene receptor
0,2
0,2
0,1
0,1
0
0
0,1
0,1
66
66
90
90
114
114
120
120
123
123
-0,1
-
0,1
0
0
-0,2
-
0,2
66
66
90
90
114
114
120
120
123
123
-0,3
-
0,3
-0,1
-
0,1
-0,4
-
0,4
-0,2
-
0,2
-0,5
-
0,5
-0,6
-
0,6
-0,3
-
0,3
-0,7
-
0,7
-0,4
-
0,4
-0,8
-
0,8
auxin repressed protein
auxin growth promoter protein
auxin
growth promoter protein
auxin
repressed protein
0,9
0,9
5
5
0,8
0,8
4
4
0,7
0,7
0,6
0,6
3
3
0,5
0,5
2
2
0,4
0,4
0,3
0,3
1
1
0,2
0,2
0,1
0,1
0
0
66
66
90
90
114
114
120
120
123
123
0
0
66
66
90
90
114
114
120
120
123
123
-
-
1
1
ETR
ACO
ACO
ACO
ETR
ETR
6
6
6
6
5
5
5
5
4
4
4
4
3
3
3
3
2
2
2
2
1
1
1
1
0
0
66
66
90
90
114
114
120
120
123
123
0
0
66
66
90
90
114
114
120
120
123
123
-
-
1
1
