# Supplementary material for: Use of homologous and heterologous gene expression profiling tools to characterize transcription dynamics during apple fruit maturation and ripening
Source: BMC Plant Biol. 2010 Oct 25;10:229. doi: 10.1186/1471-2229-10-229 (PMC3095317; doi:10.1186/1471-2229-10-229)
Supplement: Additional file 12 — Expression patterns for genes involved in cell wall metabolism, as determined with the HOM array. The black line indicates the control samples and the red line indicates samples treated with 1-MCP. Abbreviations: 1-MCP, 1-Methylcyclopropene. [file 1471-2229-10-229-S12.PPT]

## Slide 1
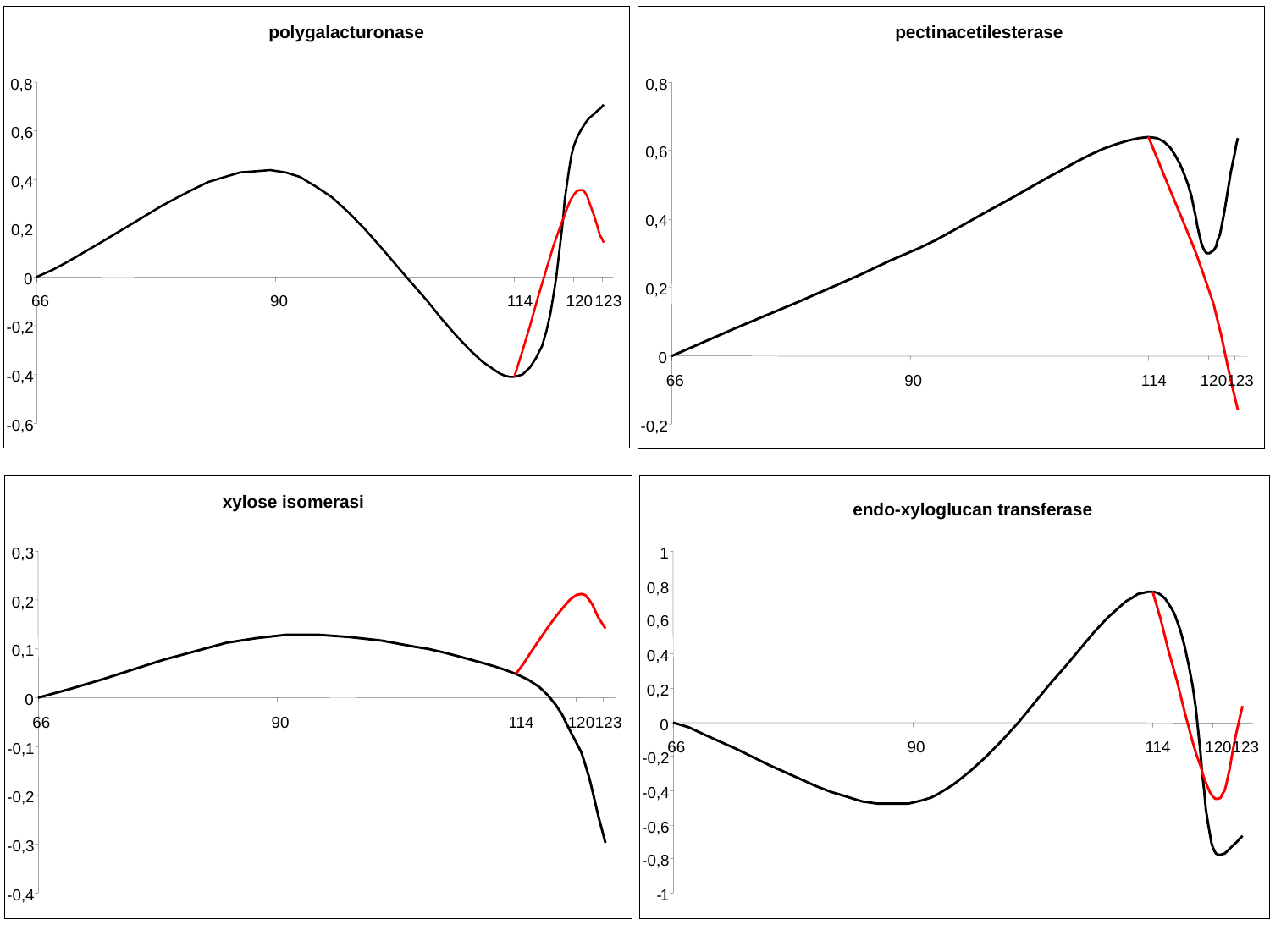

polygalacturonase
polygalacturonase
pectinacetilesterase
pectinacetilesterase
polygalacturonase
pectin acetylesterase
0,8
0,8
0,8
0,8
0,6
0,6
0,6
0,6
0,4
0,4
0,4
0,4
0,2
0,2
0
0
0,2
0,2
66
66
90
90
114
114
120
120
123
123
-0,2
-
0,2
0
0
-0,4
-
0,4
66
66
90
90
114
114
120
120
123
123
-0,6
-
0,6
-0,2
-
0,2
xylose isomerasi
xylose
isomerasi
endoxyloglucan
transferase
xylose isomerasi
endo-xyloglucan transferase
0,3
0,3
1
1
0,8
0,8
0,2
0,2
0,6
0,6
0,1
0,1
0,4
0,4
0,2
0,2
0
0
66
66
90
90
114
114
120
120
123
123
0
0
66
66
90
90
114
114
120
120
123
123
-0,1
-
0,1
-0,2
-
0,2
-0,4
-
0,4
-0,2
-
0,2
-0,6
-
0,6
-0,3
-
0,3
-0,8
-
0,8
-0,4
-
0,4
-
-
1
1
