# Supplementary material for: Use of homologous and heterologous gene expression profiling tools to characterize transcription dynamics during apple fruit maturation and ripening
Source: BMC Plant Biol. 2010 Oct 25;10:229. doi: 10.1186/1471-2229-10-229 (PMC3095317; doi:10.1186/1471-2229-10-229)
Supplement: Additional file 15 — Eighty genes (complementary to table 1) expressed during the ripening of both apple and tomato. [file 1471-2229-10-229-S15.DOC]

| **Tomato_ID** | **Apple_ID** | **Gene_annotation** |
| --- | --- | --- |
| SGN-U213861 | CN19642 | Glutamate decarboxylase (GAD) |
| SGN-U212595 | CN20282 | Histidine decarboxylase (HDC) (TOM92) |
| SGN-U212615 | CN20282 | Histidine decarboxylase (HDC) (TOM92) |
| SGN-U214486 | CN20282 | Histidine decarboxylase (HDC) (TOM92) |
| SGN-U215572 | CN26045 | serine hydroxymethyltransferase, mitochondrial precursor |
| SGN-U214286 | CN19677 | nucleoside-diphosphate-sugar epimerase/dehydratase |
| SGN-U212934 | CN9602 | histone H3.2 [Arabidopsis thaliana] |
| SGN-U212935 | CN9603 | histone H3.2 [Arabidopsis thaliana] |
| SGN-U213235 | CN4030 | histone H3 [Arabidopsis thaliana] |
| SGN-U219139 | CN22865 | formin homology 2 (FH2) domain-containing protein [Arabidopsis thaliana] |
| SGN-U228853 | CN24978 | fimbrin-like protein AtFim2 [Arabidopsis thaliana] |
| SGN-U212618 | CN3149 | beta tubulin [Oryza sativa (japonica cultivar-group)] |
| SGN-U212624 | CN3150 | beta tubulin [Arabidopsis thaliana] |
| SGN-U212647 | CN20651 | alpha-tubulin [Nicotiana tabacum] |
| SGN-U213044 | CN18657 | syringolide-induced protein 19-1-5 [Glycine max] |
| SGN-U214840 | CN27022 | cellulose synthase isolog [Arabidopsis thaliana] |
| SGN-U213451 | CN7221 | pathogenesis-related protein PR-1 precursor [Capsicum annuum] |
| SGN-U213948 | CN21519 | hypersensitive-induced response protein [Arabidopsis thaliana] |
| SGN-U227528 | CN8922 | growth regulator protein [Arabidopsis thaliana] |
| SGN-U212948 | CN19933 | arginine decarboxylase 1 [Datura stramonium] |
| SGN-U214537 | CN20039 | ACYL-COA-BINDING PROTEIN (ACBP) |
| SGN-U212783 | CN25174 | lipoxygenase [Lycopersicon esculentum] |
| SGN-U215050 | CN15107 | cytidine deaminase - like [Arabidopsis thaliana] |
| SGN-U214907 | CN9045 | arginine/serine-rich splicing factor (atSRp34), putative [Arabidopsis thaliana] |
| SGN-U213517 | CN6376 | plasma intrinsic protein 2,1 [Juglans regia] |
| SGN-U212574 | CN1062 | aldolase, plastidic [Nicotiana paniculata] |
| SGN-U225521 | CN20849 | Ribulose bisphosphate carboxylase small chain 3A/3C, chloroplast precursor |
| SGN-U225539 | CN18329 | Ribulose bisphosphate carboxylase small chain 3A/3C, chloroplast precursor |
| SGN-U225539 | CN18329 | Ribulose bisphosphate carboxylase small chain 3A/3C, chloroplast precursor |
| SGN-U225539 | CN18329 | Ribulose bisphosphate carboxylase small chain 3A/3C, chloroplast precursor |
| SGN-U225545 | CN9155 | Ribulose bisphosphate carboxylase small chain 1, chloroplast precursor |
| SGN-U212697 | CN20647 | chlorophyll a/b-binding protein type I precursor - tomato |
| SGN-U212937 | CN20139 | Chlorophyll A-B binding protein 13, chloroplast precursor |
| SGN-U212938 | CN20139 | Chlorophyll A-B binding protein 13, chloroplast precursor |
| SGN-U213105 | CN960 | chlorophyll a/b-binding protein type I precursor (cab-6A) - tomato |
| SGN-U218905 | CN20591 | Chlorophyll A-B binding protein 1B, chloroplast precursor |
| SGN-U218921 | CN20591 | Chlorophyll A-B binding protein 3C, chloroplast precursor |
| SGN-U212665 | CN139 | Photosystem II 10 kDa polypeptide, chloroplast precursor |
| SGN-U213214 | CN655 | Photosystem II core complex proteins psbY, chloroplast precursor |
| SGN-U213381 | CN1485 | photosystem I reaction center subunit X psaK [Nicotiana tabacum] |
| SGN-U213581 | CN20121 | Photosystem I reaction center subunit IV B, chloroplast precursor (PSI-E B) |
| SGN-U212743 | CN20568 | Oxygen-evolving enhancer protein 1, chloroplast precursor |
| SGN-U218772 | CN15354 | Protoporphyrinogen oxidase, mitochondrial (PPO II) |
| SGN-U212746 | CN19769 | Peptidyl-prolyl cis-trans isomerase |
| SGN-U213332 | CN19737 | peptidylprolyl isomerase (ROF1) [Arabidopsis thaliana] |
| SGN-U212554 | CN7733 | ribosomal protein L11-like [Nicotiana tabacum] |
| SGN-U212967 | CN17088 | ribosomal protein, 60S ribosomal protein L10A |
| SGN-U213207 | CN20500 | ribosomal protein, 60S ACIDIC RIBOSOMAL PROTEIN P0 |
| SGN-U213250 | CN19695 | ribosomal protein, 40S RIBOSOMAL PROTEIN S11 |
| SGN-U213269 | CN882 | ribosomal protein, 60S RIBOSOMAL PROTEIN L36 |
| SGN-U213298 | CN11820 | ribosomal protein, 60S ribosomal protein L13A |
| SGN-U213322 | CN3241 | ribosomal protein S29 [Oryza sativa (japonica cultivar-group)] |
| SGN-U213480 | CN290 | ribosomal protein S21 - like [Arabidopsis thaliana] |
| SGN-U213813 | CN19720 | ribosomal protein L1 protein |
| SGN-U214260 | CN4225 | ribosomal protein, 60S ribosomal protein L26 |
| SGN-U214351 | CN918056 | ribosomal protein, 60S RIBOSOMAL PROTEIN L30 |
| SGN-U214958 | CN15702 | ribosomal protein, 30S ribosomal protein S13, chloroplast precursor |
| SGN-U215149 | CN14342 | ribosomal protein, 60S ribosomal protein L35 [Euphorbia esula] |
| SGN-U216970 | CN534 | Cysteine proteinase 3 precursor |
| SGN-U214388 | CN5350 | proteasome subunit alpha type 7 |
| SGN-U215092 | CN21707 | ubiquitin-conjugating enzyme 9 (UBC9) [Arabidopsis thaliana] |
| SGN-U214714 | CN25680 | CBL-interacting protein kinase 1 [Arabidopsis thaliana] |
| SGN-U215637 | CN7833 | protein kinase [Arabidopsis thaliana] |
| SGN-U218369 | CN8862 | protein kinase - tomato |
| SGN-U216183 | CN27665 | mitogen-activated protein kinase 2 [Lycopersicon esculentum] |
| SGN-U218093 | CN1548 | leucine-rich repeat transmembrane protein kinase, putative [Arabidopsis thaliana] |
| SGN-U213441 | CN7084 | Serine/threonine Kinase [Persea americana] |
| SGN-U214825 | CN21823 | protein phosphatase 2A [Fagus sylvatica] |
| SGN-U215739 | CN27896 | protein phosphatase -related [Arabidopsis thaliana] |
| SGN-U220009 | CN16437 | calcineurin-like phosphoesterase family [Arabidopsis thaliana] |
| SGN-U213418 | CN6833 | phosphoprotein phosphatase (EC 3.1.3.16) 2A regulatory chain - common tobacco |
| SGN-U214209 | CN16614 | Ser/Thr specific protein phosphatase 2A B regulatory subunit beta isoform |
| SGN-U213126 | CN2194 | Calreticulin precursor |
| SGN-U216391 | CN16213 | heat shock protein 18p - common tobacco |
| SGN-U218323 | CN20218 | dnaK-type molecular chaperone hsc70-3 - tomato |
| SGN-U213103 | CN20563 | DS2 protein [Solanum tuberosum] |
| SGN-U214124 | CN29491 | vesicle transport protein SEC22, putative [Arabidopsis thaliana] |
| SGN-U213231 | DT001993 | ABC transporter [Oryza sativa (japonica cultivar-group)] |
| SGN-U216363 | CN13450 | boron transporter [Oryza sativa (japonica cultivar-group)] |
| SGN-U231972 | CN1356 | aquaporin PIP-type pTOM75 (Ripening-associated membrane protein) |
